# Analyses of clinicopathological, molecular, and prognostic associations of *KRAS* codon 61 and codon 146 mutations in colorectal cancer: cohort study and literature review

**DOI:** 10.1186/1476-4598-13-135

**Published:** 2014-05-31

**Authors:** Yu Imamura, Paul Lochhead, Mai Yamauchi, Aya Kuchiba, Zhi Rong Qian, Xiaoyun Liao, Reiko Nishihara, Seungyoun Jung, Kana Wu, Katsuhiko Nosho, Yaoyu E Wang, Shouyong Peng, Adam J Bass, Kevin M Haigis, Jeffrey A Meyerhardt, Andrew T Chan, Charles S Fuchs, Shuji Ogino

**Affiliations:** 1 Department of Medical Oncology, Dana-Farber Cancer Institute and Harvard Medical School, 450 Brookline Ave., Room M422, 02215 Boston, MA, USA; 2 Gastrointestinal Research Group, Institute of Medical Sciences, University of Aberdeen, Aberdeen, UK; 3 Department of Nutrition, Harvard School of Public Health, Boston, MA, USA; 4 Channing Division of Network Medicine, Department of Medicine, Brigham and Women’s Hospital, and Harvard Medical School, Boston, MA, USA; 5 First Department of Internal Medicine, Sapporo Medical University, Sapporo, Japan; 6 Center for Cancer Computational Biology, Dana-Farber Cancer Institute, Boston, MA, USA; 7 Cancer Program, The Broad Institute of MIT and Harvard, Cambridge, MA, USA; 8 Molecular Pathology Unit and Center for Cancer Research, Massachusetts General Hospital, Charlestown, MA, USA; 9 Division of Gastroenterology, Massachusetts General Hospital, Boston, MA, USA; 10 Department of Pathology, Brigham and Women’s Hospital, and Harvard Medical School, Boston, MA, USA; 11 Department of Epidemiology, Harvard School of Public Health, Boston, MA, USA

**Keywords:** Clinical outcome, Colon cancer, Genetic change, *RAF*, *RAS*

## Abstract

**Background:**

*KRAS* mutations in codons 12 and 13 are established predictive biomarkers for anti-EGFR therapy in colorectal cancer. Previous studies suggest that *KRAS* codon 61 and 146 mutations may also predict resistance to anti-EGFR therapy in colorectal cancer. However, clinicopathological, molecular, and prognostic features of colorectal carcinoma with *KRAS* codon 61 or 146 mutation remain unclear.

**Methods:**

We utilized a molecular pathological epidemiology database of 1267 colon and rectal cancers in the Nurse’s Health Study and the Health Professionals Follow-up Study. We examined *KRAS* mutations in codons 12, 13, 61 and 146 (assessed by pyrosequencing), in relation to clinicopathological features, and tumor molecular markers, including *BRAF* and *PIK3CA* mutations, CpG island methylator phenotype (CIMP), LINE-1 methylation, and microsatellite instability (MSI). Survival analyses were performed in 1067 *BRAF*-wild-type cancers to avoid confounding by *BRAF* mutation. Cox proportional hazards models were used to compute mortality hazard ratio, adjusting for potential confounders, including disease stage, *PIK3CA* mutation, CIMP, LINE-1 hypomethylation, and MSI.

**Results:**

*KRAS* codon 61 mutations were detected in 19 cases (1.5%), and codon 146 mutations in 40 cases (3.2%). Overall *KRAS* mutation prevalence in colorectal cancers was 40% (=505/1267). Of interest, compared to *KRAS*-wild-type, overall, *KRAS*-mutated cancers more frequently exhibited cecal location (24% vs. 12% in *KRAS*-wild-type; *P* < 0.0001), CIMP-low (49% vs. 32% in *KRAS*-wild-type; *P* < 0.0001), and *PIK3CA* mutations (24% vs. 11% in *KRAS*-wild-type; *P* < 0.0001). These trends were evident irrespective of mutated codon, though statistical power was limited for codon 61 mutants. Neither *KRAS* codon 61 nor codon 146 mutation was significantly associated with clinical outcome or prognosis in univariate or multivariate analysis [colorectal cancer-specific mortality hazard ratio (HR) = 0.81, 95% confidence interval (CI) = 0.29-2.26 for codon 61 mutation; colorectal cancer-specific mortality HR = 0.86, 95% CI = 0.42-1.78 for codon 146 mutation].

**Conclusions:**

Tumors with *KRAS* mutations in codons 61 and 146 account for an appreciable proportion (approximately 5%) of colorectal cancers, and their clinicopathological and molecular features appear generally similar to *KRAS* codon 12 or 13 mutated cancers. To further assess clinical utility of *KRAS* codon 61 and 146 testing, large-scale trials are warranted.

## Introduction

Use of Standardized Official Symbols: We use HUGO (Human Genome Organisation)-approved official symbols for genes and gene products, including BRAF; EGFR; KRAS; PIK3CA; all of which are described at http://www.genenames.org.

Colorectal cancer represents a heterogeneous group of diseases, and its molecular classification is increasingly important. Colorectal cancers can be classified using mutations in oncogenes such as *KRAS*, *BRAF* and *PIK3CA*[1]. In addition, microsatellite instability (MSI) and epigenomic instability, such as the CpG island methylator phenotype (CIMP) and LINE-1 hypomethylation, have been associated with the oncogene mutations and clinical outcomes [1-4].

Approximately 30-40% of colorectal cancers harbor *KRAS* mutations, typically in codon 12 or 13 [5-9]. Features of colorectal cancers with *KRAS* codon 12 and 13 mutations include associations with cecal location [5,8], low-level CIMP (CIMP-low) [10-14], and *PIK3CA* mutation [15-18]. *KRAS* codon 12 and 13 mutations are widely accepted as a predictive biomarker of lack of response to anti-EGFR therapy in metastatic colorectal cancer [19-23], though a few exploratory studies suggest that codon 13 mutants may benefit from EGFR-targeted therapy [24,25].

*KRAS* codons 61 and 146 are additional hotspots for mutation in colorectal cancer, and data from a small number of studies suggest that *KRAS* mutation at these sites may predict resistance to anti-EGFR therapy [26-28]. Recently, Douillard et al., utilizing existing clinical trial data, reported that *KRAS* mutations in codons 61, 146, and 117, and mutations in *NRAS*, might identify patients with metastatic colorectal cancer who fail to derive benefit from panitumumab plus FOLFOX4 [29]. Despite growing clinical relevance, the clinicopathological and molecular features of colorectal cancers with *KRAS* codon 61 or 146 mutation remain largely unknown. It is of interest to examine the characteristics of colorectal cancers with *KRAS* mutations in codons 61 and 146, compared to those in codons 12 and 13, and *KRAS*-wild-type cases. In the near future, routine clinical testing of these additional *KRAS* codons may be warranted.

We therefore investigated the clinicopathological, molecular, and prognostic characteristics of tumors harboring *KRAS* codon 61 and 146 mutations, utilizing a molecular pathological epidemiology [30,31] database of 1267 colorectal cancers from two U.S. nationwide prospective cohort studies. We also performed a comprehensive review on *KRAS* codon 61 and 146 mutations in colorectal cancer, and our curated literature data can be readily useful for public databases such as the COSMIC (Catalogue of Somatic Mutations in Cancer) database.

## Results

### *KRAS* codon 12, 13, 61 and 146 mutations, in relation to clinicopathological and molecular features

We detected *KRAS* mutations in 505 (40%) cases in 1267 colorectal cancers (Table 1). Codon 12 mutations were present in 344 cases (27%), codon 13 mutations in 115 cases (9.1%), codon 61 mutations in 19 cases (1.5%), and codon 146 mutations in 40 cases (3.2%). There were 493 cases with *KRAS* mutations identified in only one of codons 12, 13, 61 and 146, and 12 cases with *KRAS* mutations identified in two or more of the four codons (Table 1).

**Table 1:** **Frequencies of *KRAS *** **mutations in 1267 colorectal cancer cases**

**Codon**	**Nucleotide change**	**Amino acid change**	**Codon change**	**No. of cases**	**Proportion among 1267 cases**
Any	Any	Any	Any	505	40%
12	Any	Any	Any	344	27%
13	Any	Any	Any	115	9.1%
61	Any	Any	Any	19	1.5%
146	Any	Any	Any	40	3.2%
Mutations identified in only one of codons 12, 13, 61 and 146					
12	c.34G>A	p.G12S	GGT>AGT	12	1.0%
12	c.34G>C	p.G12R	GGT>CGT	7	0.6%
12	c.34G>T	p.G12C	GGT>TGT	43	3.4%
12	c.35G>A	p.G12D	GGT>GAT	157	12%
12	c.35G>C	p.G12A	GGT>GCT	20	1.6%
12	c.35G>T	p.G12V	GGT>GTT	93	7.3%
12	c.35_36delinsCA	p.G12A	GGT>GCA	1	0.1%
13	c.37G>C	p.G13R	GGC>CGC	1	0.1%
13	c.37G>T	p.G13C	GGC>TGC	2	0.2%
13	c.38G>A	p.G13D	GGC>GAC	103	8.1%
13	c.38G>T	p.G13V	GGC>GTC	2	0.2%
61	c.182A>G	p.Q61R	CAA>CGA	2	0.2%
61	c.182A>T	p.Q61L	CAA>CTA	4	0.3%
61	c.183A>C	p.Q61H	CAA>CAC	7	0.6%
60, 61	c.180_181delinsAA	p.Q61K	GGT + CAA>GGA + AAA	4	0.3%
146	c.436G>A	p.A146T	GCA>ACA	21	1.7%
146	c.436G>C	p.A146P	GCA>CCA	3	0.2%
146	c.437C>T	p.A146V	GCA>GTA	11	0.9%
Mutations identified in two or more of codons 12, 13, 61 and 146					
12, 13	c.35G>A, c.38G>A	p.G12D, p.G13D	GGT>GAT, GGC>GAC	4	0.3%
12, 13	c.35G>T, c.37G>T	p.G12V, p.G13C	GGT>GTT, GGC>TGC	1	0.1%
12,	c.35G>T,	p.G12V,	GGT>GTT, GGT + CAA>GGA + AAA	1	0.1%
60, 61	c.180_181delinsAA	p.Q61K			
12, 146	c.34G>C, c.436G>A	p.G12R, p.A146T	GGT>CGT, GCA>ACA	1	0.1%
12, 146	c.34G>T, c.436G>A	p.G12C, p.A146T	GGT>TGT, GCA>ACA	1	0.1%
12, 146	c.34G>T, c.437C>T	p.G12C, p.A146V	GGT>TGT, GCA>GTA	1	0.1%
12, 146	c.35G>T, c.436G>A	p.G12V, p.A146T	GGT>GTT, GCA>ACA	1	0.1%
13, 146	c.38G>A, c.436G>A	p.G13D, p.A146T	GGC>GAC, GCA>ACA	1	0.1%
12, 13, 61	c.35G>A, c.38G>A, c.183A>T	p.G12D, p.G13D, p.Q61H	GGT>GAT, GGC>GAC, CAA>CAT	1	0.1%

The baseline characteristics of study subjects are summarized in Table 2, according to tumor *KRAS* mutation status. Compared to *KRAS*-wild-type tumors, overall *KRAS*-mutated cancers were less likely to exhibit poor differentiation (5.8%, *P* < 0.0001), MSI-high (6.2%, *P* < 0.0001), and *BRAF* mutation (1.4%, *P* < 0.0001), and more likely to demonstrate cecal location (24%, *P* < 0.0001), CIMP-low (49%, *P* < 0.0001), and *PIK3CA* mutation (24%, *P* < 0.0001). Of note, these trends were generally evident across case groups with specific mutated codons (Table 2). *KRAS* mutation status was not significantly associated with sex, age, body mass index (BMI), year of diagnosis, family history of colorectal cancer, disease stage, peritumoral lymphocytic reaction, or tumor LINE-1 methylation level. There was no significant difference in any of the features between the cases with *KRAS* mutations identified in only one codon (*N* = 493) and those with *KRAS* mutations identified in two or more codons (*N* = 12), though statistical power was limited, given only 12 cases with *KRAS* mutations identified in multiple codons (Additional file 1: Table S1).

**Table 2:** **Clinicopathological, and molecular characteristics according to *KRAS *** **mutation status in 1267 colorectal cancer cases**

**Clinicopathological or molecular feature**	**Total No.**	***KRAS***		***P *** **(Wild-type vs. mutant)**	***KRAS *** **mutations identified in only one codon**				***P *** **(Across four mutants)**
		**Wild-type**	**Mutant**		**Codon 12**	**Codon 13**	**Codon 61**	**Codon 146**	
Total No. of patients	1267	762	505		333	108	17	35	
Sex				0.0091					0.11
Male	573 (45%)	322 (42%)	251 (50%)		162 (49%)	59 (55%)	4 (24%)	19 (54%)	
Female	694 (55%)	440 (58%)	254 (50%)		171 (51%)	49 (45%)	13 (76%)	16 (46%)	
Mean age (years) ± SD	68.6 ± 8.7	68.4 ± 8.6	68.8 ± 8.8	0.47	69.5 ± 8.5	67.5 ± 9.2	70.0 ± 9.3	66.0 ± 9.8	0.065
BMI (kg/m^2^)				0.13					0.43
<30	1025 (81%)	607 (80%)	418 (83%)		278 (84%)	88 (81%)	11 (69%)	30 (86%)	
≥30	240 (19%)	155 (20%)	85 (17%)		54 (16%)	20 (19%)	5 (31%)	5 (14%)	
Year of diagnosis				0.26					0.032
Prior to 1998	640 (51%)	375 (49%)	265 (52%)		164 (49%)	63 (58%)	5 (29%)	23 (66%)	
1998 - 2006	627 (49%)	387 (51%)	240 (48%)		169 (51%)	45 (42%)	12 (71%)	12 (34%)	
Family history of colorectal cancer in first degree relative(s)				0.76					0.87
Absent	1026 (81%)	612 (80%)	414 (82%)		273 (82%)	89 (82%)	14 (82%)	27 (77%)	
Present in one first degree relative	179 (14%)	111 (15%)	68 (13%)		44 (13%)	15 (14%)	3 (18%)	5 (14%)	
Present in two or more first degree relatives	62 (5%)	39 (5%)	23 (5%)		16 (5%)	4 (4%)	0	3 (9%)	
Tumor location				<0.0001					0.50
Cecum	209 (17%)	90 (12%)	119 (24%)		79 (24%)	27 (25%)	4 (24%)	6 (18%)	
Ascending colon	262 (21%)	171 (23%)	91 (18%)		52 (16%)	25 (24%)	3 (18%)	7 (21%)	
Hepatic flexure to transverse colon	117 (9%)	78 (10%)	39 (8%)		26 (8%)	7 (6%)	4 (24%)	2 (5%)	
Splenic flexure to descending colon	90 (7%)	57 (8%)	33 (6%)		22 (7%)	7 (6%)	0	3 (8%)	
Sigmoid colon	297 (24%)	182 (24%)	115 (23%)		83 (25%)	22 (20%)	1 (5%)	8 (24%)	
Rectum	279 (22%)	176 (23%)	103 (21%)		67 (20%)	20 (19%)	5 (29%)	8 (24%)	
Disease stage				0.028					0.89
I	298 (23%)	190 (25%)	108 (21%)		77 (23%)	20 (19%)	4 (23%)	4 (11%)	
II	354 (28%)	230 (30%)	124 (25%)		77 (23%)	30 (28%)	5 (29%)	11 (32%)	
III	328 (26%)	183 (24%)	145 (29%)		97 (29%)	29 (27%)	3 (18%)	11 (32%)	
IV	173 (14%)	93 (12%)	80 (16%)		51 (15%)	18 (16%)	2 (12%)	6 (17%)	
Unknown	114 (9%)	66 (9%)	48 (9%)		31 (10%)	11 (10%)	3 (18%)	3 (8%)	
Tumor differentiation				<0.0001					0.55
Well-moderate	1137 (90%)	663 (88%)	474 (94%)		314 (95%)	99 (92%)	16 (94%)	34 (97%)	
Poor	123 (10%)	94 (12%)	29 (6%)		17 (5%)	9 (8%)	1 (6%)	1 (3%)	
Peritumoral lymphocytic reaction				0.042					0.48
Absent-minimal	164 (14%)	96 (13%)	68 (14%)		47 (15%)	14 (13%)	2 (12%)	4 (12%)	
Mild	878 (72%)	515 (71%)	363 (75%)		237 (75%)	76 (71%)	12 (76%)	28 (85%)	
Moderate-marked	170 (14%)	117 (16%)	53 (11%)		32 (10%)	17 (16%)	2 (12%)	1 (3%)	
MSI status				<0.0001					0.078
MSI-low/MSS	1057 (85%)	587 (79%)	470 (94%)		315 (95%)	100 (94%)	14 (82%)	31 (89%)	
MSI-high	191 (15%)	160 (21%)	31 (6.2%)		16 (4.8%)	6 (5.7%)	3 (18%)	4 (11%)	
CIMP status				<0.0001					0.014
CIMP-negative	521 (44%)	311 (44%)	210 (44%)		139 (44%)	37 (36%)	8 (50%)	19 (54%)	
CIMP-low	460 (39%)	224 (32%)	236 (49%)		154 (49%)	59 (57%)	4 (25%)	16 (46%)	
CIMP-high	206 (17%)	172 (24%)	34 (7%)		21 (7%)	7 (7%)	4 (25%)	0	
*PIK3CA* mutation status				<0.0001					0.63
Wild-type	983 (84%)	632 (89%)	351 (76%)		242 (78%)	72 (74%)	12 (80%)	19 (68%)	
Mutant	190 (16%)	78 (11%)	112 (24%)		70 (22%)	25 (26%)	3 (20%)	9 (32%)	
*BRAF* mutation status				<0.0001					0.25
Wild-type	1078 (85%)	582 (77%)	496 (99%)		328 (99%)	106 (98%)	16 (94%)	35 (100%)	
Mutant	184 (15%)	177 (23%)	7 (1%)		3 (1%)	2 (2%)	1 (6%)	0	
Mean LINE-1 methylation level (%) ± SD	62.7 ± 9.3	62.8 ± 9.6	62.5 ± 9.0	0.33	62.7 ± 9.2	61.5 ± 8.2	64.2 ± 10.1	63.1 ± 9.0	0.42

(%) indicates the proportion of cases with a specific clinicopathological, or molecular feature among each *KRAS* mutation status group. The *P*-value for significance was adjusted for multiple hypothesis testing to *P* = 0.05/14 = 0.0036. Thus, a *P*-value between 0.05 and 0.0036 should be regarded as of borderline significance. BMI, body mass index; CIMP, CpG island methylator phenotype; MSI, microsatellite instability; MSS, microsatellite stable; SD, standard deviation.

### *KRAS* mutation status and patient survival in *BRAF*-wild-type cases

To examine the prognostic role of *KRAS* mutation independent of *BRAF* mutation, within 1067 *BRAF*-wild-type cases (excluding *BRAF* mutants), we compared *KRAS*-mutated cancers to cases with wild-type *KRAS* in all four codons 12, 13, 61 and 146 (Additional file 2: Table S2). We evaluated clinicopathological, molecular and survival data of 51 cases with *KRAS* codon 61 and 146 mutations (Additional file 3: Table S3). There were 514 deaths, including 307 colorectal cancer-specific deaths, during a median follow-up of 11.7 years (interquartile range, 8.3-16.1 years) for censored cases.

**Figure 1:**
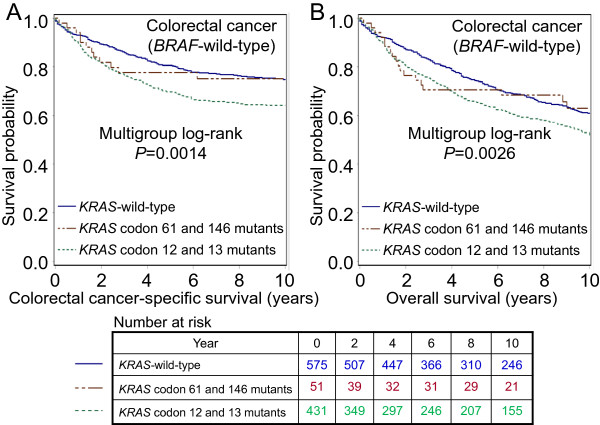
**Kaplan-Meier curves for colorectal cancer patients with *BRAF-*wild-type tumors, according to tumor *KRAS *mutation status. (A)** Colorectal cancer-specific survival. **(B)** Overall survival. Table indicates the number of patients who were alive and at risk of death at each time point after diagnosis of colorectal cancer.

The 5-year colorectal cancer-specific survival probabilities were 80.6% for cases with *KRAS*-wild-type/*BRAF*-wild-type tumors, 67.9% for cases with codon 12 mutations, 75.8% for cases with codon 13 mutations, 79.4% for cases with codon 61 mutations, and 76.7% for cases with codon 146 mutations. Specific *KRAS* mutations were significantly associated with patient survival in Kaplan-Meier analysis (log-rank *P* = 0.0014, Figure 1). In multivariate analysis, compared to *KRAS*-wild-type/*BRAF*-wild-type tumors, we observed a significant prognostic association for *KRAS* codon 12 mutation [multivariate hazard ratio (HR) = 1.45; 95% confidence interval (CI), 1.12-1.87; *P* = 0.0048; Table 3). However, neither mutation of *KRAS* codon 61 nor codon 146 was associated with patient outcome (Table 3). For cases with the 10 most common *KRAS* mutations across all four codons examined, those with the c.34G>T (p.G12C) mutation, and those with the c.35G>T (p.G12V) mutation experienced significantly higher colorectal cancer-specific mortality in Cox regression analysis [multivariate HR = 2.33; 95% CI, 1.36-3.99; *P* = 0.0021 for c.34G>T (p.G12C); multivariate HR = 2.13; 95% CI, 1.47-3.09; *P* < 0.0001 for c.35G>T (p.G12V); Table 3], even after adjusting a statistical significance level for multiple testing (*P* < 0.005). None of the three most common *KRAS* mutations in codons 61 and 146 [c.183A>C (p.Q61H), c.436G>A (p.A146T) and c.437C>T (p.A146V)] was associated with patient prognosis (Table 3), although statistical power was limited. Subgroup analyses of stage I-II cases (*N* = 544, Additional file 4: Table S4), and stage III-IV cases (*N* = 414, Additional file 5: Table S5) yielded similar results, although statistical power was limited.

**Table 3:** **Colorectal cancer patient mortality according to *KRAS *** **mutation status in 1067 *BRAF*-wild-type cases**

***KRAS***	**Total No.**	**Colorectal cancer-specific mortality**			**Overall mortality**		
		**No. of events**	**Univariate HR (95% CI)**	**Multivariate stage-stratified HR (95% CI)**	**No. of events**	**Univariate HR (95% CI)**	**Multivariate stage-stratified HR (95% CI)**
Wild-type (codons 12, 13, 61 and 146)	582	144	1 (referent)	1 (referent)	258	1 (referent)	1 (referent)
All mutants together	485	163	1.46 (1.17-1.83)	1.19 (0.94-1.51)	256	1.32 (1.11-1.57)	1.14 (0.95-1.38)
			*P* = 0.0009			*P* = 0.0018	
Codons 12 and 13, and codons 61 and 146							
Codons 12 and 13	434	151	1.51 (1.20-1.90)	1.27 (0.99-1.62)	235	1.36 (1.14-1.62)	1.15 (0.95-1.40)
			*P* = 0.0004			*P* = 0.0007	
Codons 61 and 146	51	12	1.02 (0.57-1.85)	0.85 (0.47-1.56)	21	1.00 (0.64-1.56)	1.07 (0.68-1.68)
Codons 12, 13, 61 and 146							
Codon 12 mutants	328	121	1.64 (1.29-2.09)	1.45 (1.12-1.87)	183	1.45 (1.20-1.76)	1.24 (1.01-1.52)
			*P* < 0.0001	*P* = 0.0048		*P* = 0.0001	*P* = 0.037
Codon 13 mutants	106	30	1.16 (0.78-1.72)	0.83 (0.55-1.25)	52	1.11 (0.82-1.49)	0.90 (0.66-1.24)
Codon 61 mutants	16	4	1.11 (0.41-3.01)	0.81 (0.29-2.26)	8	1.43 (0.71-2.90)	1.55 (0.75-3.18)
Codon 146 mutants	35	8	0.98 (0.48-2.01)	0.86 (0.42-1.78)	13	0.84 (0.48-1.48)	0.88 (0.50-1.56)
The 10 most common mutations in codons 12, 13, 61 and 146							
c.34G>A (p.G12S)	12	6	2.44 (1.07-5.54)	0.94 (0.39-2.23)	7	1.57 (0.74-3.33)	0.77 (0.35-1.70)
			*P* = 0.033				
c.34G>C (p.G12R)	7	5	5.25 (2.13-12.9)	3.44 (1.25-9.43)	6	4.69 (2.06-10.6)	3.51 (1.42-8.70)
			*P* = 0.0003	*P* = 0.017		*P* = 0.0002	*P* = 0.0067
c.34G>T (p.G12C)	42	16	1.70 (1.01-2.86)	2.33 (1.36-3.99)	25	1.56 (1.03-2.35)	1.57 (1.02-2.42)
			*P* = 0.044	*P* = 0.0021		*P* = 0.035	*P* = 0.039
c.35G>A (p.G12D)	155	51	1.46 (1.06-2.01)	1.18 (0.84-1.66)	80	1.37 (1.06-1.76)	1.16 (0.89-1.51)
			*P* = 0.021			*P* = 0.015	
c.35G>C (p.G12A)	19	6	1.28 (0.56-2.90)	0.61 (0.26-1.42)	9	1.00 (0.51-1.95)	0.59 (0.30-1.17)
c.35G>T (p.G12V)	92	37	1.76 (1.22-2.52)	2.13 (1.47-3.09)	56	1.54 (1.16-2.06)	1.54 (1.14-2.08)
			*P* = 0.0024	*P* < 0.0001		*P* = 0.0033	*P* = 0.0048
c.38G>A (p.G13D)	101	30	1.23 (0.83-1.82)	0.83 (0.55-1.26)	50	1.14 (0.84-1.54)	0.91 (0.66-1.25)
c.183A>C (p.Q61H)	7	2	1.06 (0.26-4.28)	0.46 (0.11-1.93)	4	1.28 (0.48-3.45)	1.16 (0.42-3.18)
c.436G>A (p.A146T)	21	3	0.55 (0.17-1.71)	0.50 (0.16-1.59)	5	0.45 (0.19-1.10)	0.51 (0.21-1.26)
c.437C>T (p.A146V)	11	4	1.94 (0.72-5.26)	1.77 (0.64-4.90)	7	2.02 (0.95-4.29)	2.10 (0.97-4.56)

The multivariate, stage-stratified Cox regression model initially included sex, age, body mass index, year of diagnosis, family history of colorectal cancer, tumor location, tumor differentiation, peritumoral lymphocytic reaction, microsatellite instability, CpG island methylator phenotype, *PIK3CA* mutation, and LINE-1 methylation. A backward elimination with a threshold of *P* = 0.20 was used to select variables in the final model.

For the survival analysis of mutations in the two groups of *KRAS* codons (codons 12 and 13, and codons 61 and 146), the *P*-value for significance was adjusted for multiple hypothesis testing to *P* = 0.05/2 = 0.025. Thus, a *P*-value between 0.05 and 0.025 should be regarded as of borderline significance. For the survival analysis of mutations in the four *KRAS* codons (12, 13, 61 and 146), the *P*-value for significance was adjusted for multiple hypothesis testing to *P* = 0.05/4 = 0.013. Thus, a *P*-value between 0.05 and 0.013 should be regarded as of borderline significance. For the survival analysis of the 10 most common *KRAS* mutations, the *P*-value for significance was adjusted for multiple hypothesis testing to *P* = 0.05/10 = 0.005. Thus, a *P*-value between 0.05 and 0.005 should be regarded as of borderline significance. CI, confidence interval; HR, hazard ratio.

## Discussion

Although a number of studies have examined codon 61 or 146 hotspot mutations in colorectal cancer (Additional file 6: Table S6) [26-29,32-74], clinicopathological, molecular, and prognostic characteristics of those mutations have not been well investigated. Our data, from 1267 tumors, suggest that approximately 5% of all colorectal cancers harbor *KRAS* mutations in codon 61 or 146, and those colorectal cancers generally show similar characteristics to tumors with *KRAS* mutations in codon 12 or 13 (including associations with cecal location, CIMP-low and *PIK3CA* mutations).

A variety of methods have been used for *KRAS* codon 61 and 146 analyses (Additional file 6: Table S6) [26-29,32-74], which might have contributed to a wide variation in the prevalence of those mutations. Generally, nonsequencing methods make it cumbersome to confirm multiple independent mutations, and make it difficult to detect multiple variations at one allele without employing an expanded panel of probes or primers. Of the sequencing-based methodologies, pyrosequencing has been shown to be more sensitive than Sanger sequencing in paraffin-embedded archival tissue, with the capacity to reliably detect mutant alleles at low abundance (5-10% mutant), which is common in solid tumors [75].

The association between cecal cancers and *KRAS* mutations is intriguing. Emerging data suggest that gut luminal contents and microbiota, which change along bowel subsites, play important roles in colorectal carcinogenesis [8,76]. Our recent study on colorectal cancers in detailed subsites (from cecum to rectum) has shown that tumor molecular features (including *BRAF* mutation, MSI and CIMP-high) change along the bowel subsites, and that cecal cancers are associated with *KRAS* codon 12 and 13 mutations [5,8]. In our current study, cecal cancers appeared to be significantly associated with overall *KRAS* mutation status, and this trend was evident across all four mutated codons. Further studies are needed to elucidate why *KRAS* mutations, irrespective of mutated codon, are particularly common in cecal cancers.

Examining associations of tumor molecular features can provide insights into carcinogenesis processes, and is important in cancer research [77-83]. Previous studies have demonstrated that *KRAS* codon 12 and 13 mutations are associated with aberrant DNA methylation patterns, namely CIMP-low [10,11]. Our current study suggests that *KRAS* mutation, irrespective of mutated codon (statistical power was limited for codon 61 mutants), is associated with CIMP-low. It remains to be investigated why *KRAS* mutations are associated with CIMP-low in colorectal cancer. *KRAS* have been positively associated with *PIK3CA* mutations in colorectal cancer [15-18]. Our data suggest that *KRAS* mutations, irrespective of mutated codon, are associated with *PIK3CA* mutations. It has been reported that activated RAS signaling potentiates PI3K (phosphatidylinositol-4,5-bisphosphonate 3-kinase)/AKT signaling, which is augmented by the presence of *PIK3CA* mutations [84]. Considering a possible role for *PIK3CA* mutation as a predictive biomarker of response to adjuvant aspirin therapy in colorectal cancer [16], our finding may be of interest. *KRAS* codon 12 and 13 mutations have been inversely associated with *BRAF* mutation in colorectal cancer [17,26,33,41]. Our current data suggest that *KRAS* mutations, irrespective of mutated codon, are inversely associated with MSI-high and *BRAF* mutations in colorectal cancer. LINE-1 methylation level is a surrogate marker for global DNA methylation, and has been reported to be associated with MSI-high and CIMP-high in colorectal cancer [85]. This study showed that LINE-1 methylation level in average did not significantly differ according to *KRAS* mutation status.

Experimental studies are consistent with our observations that both *KRAS* codon 61 and 146 mutations can contribute to carcinogenesis in a similar manner to oncogenic mutations in codons 12 and 13. As *KRAS* codon 12 and 13 mutations, codon 61 mutation results in oncogenic RAS with impaired GTPase activity, resulting in constitutive activation [86,87]. *KRAS* codon 146 mutation-transfected HEK-293FT cells showed a larger amount of RAS-GTP compared to *KRAS*-wild-type-transfected cells [28]. These experimental data provide an insights into plausible functional roles of codon 61 and 146 mutations in carcinogenesis.

In our current survival analysis, there was no significant association between *KRAS* codon 61 and 146 mutations, and patient outcome. The prognostic value of *KRAS* mutation in colorectal cancer remains controversial [7,88-92]. Of note, in our current study, when we separately examined specific *KRAS* mutations, codon 12 mutations [especially c.34G>T (p.G12C) and c.35G>T (p.G12V)] were significantly associated with inferior survival, which is consistent with the ‘RASCAL II’ meta-analysis [88]. Accordingly, the prognostic associations of *KRAS* mutations in colorectal cancer may vary by specific mutation. Considered in conjunction with evidence that *KRAS* codon 61 and 146 mutations possess weaker transforming potential than codon 12 mutations [40], it may be the case that *KRAS* codon 61 or 146 mutation is not associated with patient prognosis. However, considering the limited case and event numbers for *KRAS* codon 61 and 146 mutations, our survival analyses should be considered exploratory. Additional larger studies, perhaps necessitating pooling of data, are required to definitively assess the prognostic roles codon 61 and 146 mutations in colorectal cancer.

Several studies have examined the predictive value of *KRAS* mutation in codon 61 and/or 146 in metastatic colorectal cancer treated with anti-EGFR therapy (cetuximab or panitumumab) [26-28,41,43]. Pentheroudakis et al. did not observe any association between *KRAS* codon 61 or 146 mutation (*N* =11) and survival [41]. De Roock et al. showed that *KRAS* mutation in codon 61 (*N* =13), but not that in codon 146 (*N* =11), was significantly associated with lack of response to cetuximab [27]. Seymour et al. reported that *KRAS* codon 146 mutations (*N* =17) were not associated with overall or progression-free survival [43]. In contrast, Loupakis et al. reported that, among *BRAF*-wild-type cancers, *KRAS* codon 61 or 146 mutant cases (*N* = 8) experienced a significantly lower response rate and progression-free survival [26]. Indeed, a few experimental studies also reported that tumors harboring *KRAS* mutations in codons 61 and 146 were resistant to anti-EGFR therapy [28,93]. In addition, a recent published study reported by Douillard et al., showed that *RAS* mutants (*N* = 108) with any mutation in *KRAS* codons 61, 117 and 146, or *NRAS* codons 12, 13, 61, 117 and 146, did not benefit from combined panitumumab plus FOLFOX4 chemotherapy [29]. In our dataset, due to scarcity of data on cancer treatment, we were unable to examine the important question of the predictive value of *KRAS* mutations in relation to anti-EGFR therapy. Further clinical studies in this area are clearly required.

The question arises as to whether it is worth investigating these relatively rare mutations in the clinical setting. Given that over 250,000 individuals each year die of colorectal cancer in Europe and the U.S., and most of these unfavorable outcomes are due to distant metastases, we estimate that every year approximately 10,000 cases have *KRAS* mutations in codon 61 or 146, and would be regarded as *KRAS*-wild-type through current *KRAS* codon 12 and 13 testing protocols. Considering that *KRAS* codon 61 and 146 mutations may also confer resistance to EGFR inhibitors [26-29,93], patients who have metastatic colorectal cancer with *KRAS* mutation in codon 61 or 146 could receive more tailored management through clinical testing of these additional *KRAS* codons.

A limitation of this study is the absence of data on *KRAS* codon 117 mutation and *NRAS* mutations. As a result, we could not refine purer *RAS*-wild-type (both *KRAS*- and *NRAS*-wild-type in codons 12, 13, 61, 117 and 146), or examine clinicopathological, molecular and prognostic features of those whole *RAS* mutations in this study. Considering that *RAS* mutations in those codons have been reported to predict lack of response to anti-EGFR therapy in colorectal cancer [29], further studies are necessary to answer important questions about features across various *RAS* mutants. Nonetheless, *KRAS* codons 61 and 146 are the most frequent mutational hotspots after *KRAS* codons 12 and 13. In addition, our current analysis (N>1200) represents a large single study to date (Additional file 6: Table S6) [26-29,32-74], examining *KRAS* codon 61 and 146 mutations, in relation to other important molecular features in colorectal cancers, such as status of CIMP, MSI, *BRAF* and *PIK3CA* mutations. Sample size is a critical issue when assessing these relatively infrequent mutations. Indeed, smaller studies (N < 300, Additional file 6: Table S6) demonstrate considerable variability in the frequencies and distribution of reported *KRAS* mutations, ranging from 0.4% to 9.3% for *KRAS* codon 61 mutations, and from 1.3% to 6.6% for *KRAS* codon 146 mutations (Additional file 6: Table S6) [26-29,32-74]. Given the relatively low frequencies of these mutations, a large sample size is a prerequisite for assessing the prevalence of these mutations and their associations with other tumor molecular characteristics.

There are advantages in utilizing the molecular pathological epidemiology [30,31] database of the two U.S. nationwide prospective cohort studies to assess prevalence and associations of *KRAS* codon 61 and 146 mutations. Selection bias is an inevitable issue when analyzing cases identified from a few academic hospitals, since patients have selected hospitals based on referral, health insurance applicability, and/or their own preference. In contrast, a large population-based or multicenter study is desirable to decrease the degree of such selection bias. In this study, cohort participants who were diagnosed with colorectal cancer were treated at hospitals throughout the U.S., and thus constitute a more representative sample of colorectal cancers in the U.S. population than patients in a few academic hospitals.

## Conclusions

Our data from over 1200 colorectal cancers demonstrate that *KRAS* codon 61 or 146 hotspot mutations are present in approximately up to 5% of colorectal cancers, and those cancers exhibit similar clinicopathological and molecular features to cancers with *KRAS* codon 12 or 13 mutation. Our current findings suggest that additional large-scale studies are warranted to assess clinical utility of *KRAS* codon 61 and 146 testing in colorectal cancer.

## Materials and methods

### Study population

**Figure 2:**
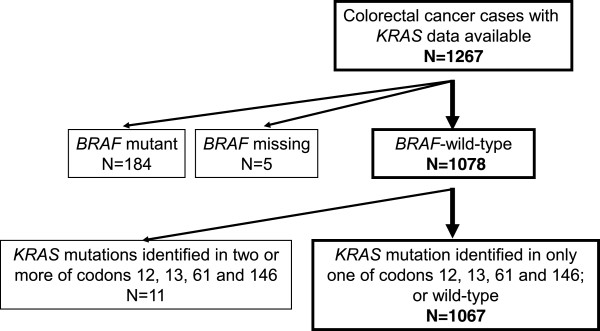
**Flow chart of the current study.** Cases with *BRAF* mutation (N = 184) and those without available *BRAF* mutation data (N = 5), were excluded from survival analyses. In addition, cases with *KRAS* mutations identified in two or more of codons 12, 13, 61 and 146 (N = 11) were excluded, in order to assess a prognostic effect of specific *KRAS* mutations individually.

We utilized two prospective cohort studies, the Nurses’ Health Study (*N* = 121,701 women followed since 1976) and the Health Professionals Follow-up Study (*N* = 51,529 men followed since 1986) [16]. Every two years, cohort participants have been sent follow-up questionnaires to identify newly diagnosed cancers in themselves and their first degree relatives. The National Death Index was used to ascertain deaths of participants as well as unreported lethal cancers. The cause of death was assigned by study physicians. Formalin-fixed paraffin-embedded tissue blocks were collected from hospitals where participants with colorectal cancer had undergone colorectal resection or diagnostic biopsy (for preoperatively-treated rectal cancers). We used 1267 colorectal cancer cases, diagnosed up to 2006, based on the availability of *KRAS* sequencing data. In order to examine the prognostic role of specific *KRAS* mutations, independent of *BRAF* mutation, *BRAF*-mutated cancers (N = 184), cases with missing *BRAF* mutation status (N = 5), and tumors with *KRAS* mutations identified in two or more of codons 12, 13, 61 and 146 (N = 11) were excluded. As a result, a final total of 1067 *BRAF*-wild-type cases were used for survival analyses (Figure 2, Additional file 2: Table S2). Informed consent was obtained from all study subjects. This study was approved by the Human Subjects Committees at Harvard School of Public Health and Brigham and Women’s Hospital. All clinicopathological and molecular analyses were performed blinded to other data, including patient outcome.

### Histopathological evaluation

Hematoxylin and eosin-stained sections of all cases were examined by a pathologist (SO) unaware of other data. Tumor differentiation was categorized as well-moderate or poor (>50% vs. ≤50% gland formation). Peritumoral lymphocytic reaction was examined as previously described [94].

### Sequencing of *KRAS* codons 61 and 146

DNA was extracted from paraffin embedded tissue as previously described, [16] and polymerase chain reaction (PCR) and pyrosequencing, targeted for *KRAS* codons 61 and 146, were performed. The PCR primers for amplifying *KRAS* codon 61 were, 5′-biotin-TGGAGAAACCTGTCTCTTGGATAT-3′ (for forward primer), and 5′-TACTGGTCCCTCATTGCACTGTA-3′ (for reverse primer), and those for *KRAS* codon 146 were 5′-ATGGAATTCCTTTTATTGAAACATC-3′ (for forward primer), and 5′-biotin-TTGCAGAAAACAGATCTGTATTTAT-3′(for reverse primer). The sequencing primers were 5′-TCATTGCACTGTACTCCTC-3′ (for codon 61), and 5′-AATTCCTTTTATTGAAACATCA-3′ (for codon 146). Dispensation orders were designed such that all possible mutations would be detected (Additional file 7: Figure S1). All mutations were confirmed by replicate analysis.

### Sequencing of *KRAS* codons 12 and 13, *BRAF,* and *PIK3CA*, and MSI analysis

We performed PCR and pyrosequencing targeted for *KRAS* (codons 12 and 13) [75], *BRAF* (codon 600) and *PIK3CA* (exons 9 and 20) as previously described [16]. MSI analysis was performed using 10 microsatellite markers (D2S123, D5S346, D17S250, BAT25, BAT26, BAT40, D18S55, D18S56, D18S67 and D18S487) [8]. MSI-high was defined as instability in ≥30% of the markers. MSI-low (<30% unstable markers) tumors were grouped with microsatellite stable (MSS) tumors (no unstable markers) because we have previously demonstrated that these two groups show similar features [8].

### Methylation analyses for CpG islands and LINE-1

Using validated bisulfite DNA treatment and real-time PCR (MethyLight), we quantified DNA methylation in eight CIMP-specific promoters [*CACNA1G*, *CDKN2A* (p16), *CRABP1*, *IGF2*, *MLH1*, *NEUROG1*, *RUNX3* and *SOCS1*] [8]. CIMP-high was defined as the presence of ≥6/8 methylated promoters, CIMP-low as 1-5/8 methylated promoters, and CIMP-negative as the absence of methylated promoters, according to established criteria [8]. In order to accurately quantify LINE-1 methylation levels, we used bisulfite pyrosequencing as previously described [8].

### Statistical analysis

All statistical analyses were performed using SAS (Version 9.2, SAS Institute, Cary, NC). All *P*-values were two-sided. Univariate analyses were performed to investigate clinicopathological and molecular characteristics according to *KRAS* mutation status; a chi-square test or Fisher’s exact test was used for categorical data, while a Wilcoxon or Kruskal-Wallis test was applied to continuous data (age and LINE-1 methylation). To account for multiple hypothesis testing in associations between *KRAS* mutation and other 14 covariates, the *P*-value for significance was adjusted by Bonferroni correction to *P* = 0.0036 (=0.05/14).

The Kaplan-Meier method and log-rank test were used to estimate survival distribution according to *KRAS* mutation status. Cases were observed until death, or January 1st 2011, whichever came first. For analyses of colorectal cancer-specific mortality, deaths as a result of other causes were censored. Cox proportional hazards regression models were used to compute mortality HRs for specific *KRAS* mutations. A multivariate model initially included the following clinicopathological and molecular variables with less than 10% of patients showing missing information among those we have previously published; sex, age (continuous), BMI (<30 vs. ≥30 kg/m^2^), year of diagnosis (continuous), family history of colorectal cancer in any first-degree relative (present vs. absent), tumor location (cecum vs. ascending colon to sigmoid colon vs. rectum), tumor differentiation (well-moderate vs. poor), peritumoral lymphocytic reaction (absent-minimal vs. mild-marked), MSI (high vs. low/MSS), CIMP (high vs. low vs. negative), *PIK3CA* mutation (present vs. absent) and LINE-1 methylation (continuous), with stratification by disease stage (I, II, III, IV or unknown) was performed using the “strata” option in the SAS “proc phreg” command. A backward elimination was performed with a threshold of *P* = 0.20, to avoid overfitting. Cases with missing information for any of the categorical covariates [BMI (0.2%), tumor location (1.0%), tumor differentiation (0.7%), peritumoral lymphocytic reaction (4.6%), MSI (1.6%), CIMP (6.7%), and *PIK3CA* (7.6%)], were included in the majority category of the given covariate to avoid overfitting. We confirmed that excluding cases with missing information in any of the covariates did not substantially alter results (data not shown). To account for multiple hypothesis testing in associations between *KRAS* mutations and patient outcome, the *P*-value for significance was adjusted by Bonferroni correction to *P* = 0.025 [=0.05/2, for the two groups of codons (codons 12 and 13, and codons 61 and 146)], *P* = 0.013 (=0.05/4, for the four codons), or *P* = 0.005 (=0.05/10, for the 10 most common mutations). The proportionality of hazards assumption was satisfied by evaluating time-dependent variables, which were the cross products of the *KRAS* indicator variables and survival time (all *P-*values>0.07).

### Literature search

A systematic literature search was performed in Pubmed, up to April 5, 2014, using combinations of the following search terms; *KRAS*, codon, (61 or 146), (colon, rectal or colorectal), and (cancer, carcinoma or adenocarcinoma). All eligible publications were retrieved, and their references were checked to identify further relevant studies. In addition, we contacted some corresponding authors to obtain detailed data.

## Abbreviations

CI: Confidence interval; CIMP: CpG island methylator phenotype; HR: Hazard ratio; MSI: Microsatellite instability; MSS: Microsatellite stable; PCR: Polymerase chain reaction; PI3K: Phosphatidylinositol-4,5-bisphosphonate 3-kinase.

## Competing interests

ATC previously served as a consultant for Bayer Healthcare, Millennium Pharmaceuticals, and Pfizer Inc. This study was not funded by Bayer Healthcare, Millennium Pharmaceuticals, or Pfizer Inc. No other conflict of interest exists.

## Authors’ contributions

YI, SO and KMH conceived of the study. YI, PL, MY, ZRQ, XL and KN carried out molecular analysis. YI and SO interpreted the data and drafted the manuscript. AK, RN, SJ, KW, YEW, SP and AJB helped the statistical analysis and participated in interpretation of data. JAM, ATC and CSF helped to draft the manuscript, and participated in interpretation of data. All authors read and approved the final manuscript.

## Supplementary Material

Additional file 1: Table S1 Clinicopathological, and molecular characteristics of *KRAS*-wild-type, only-one-*KRAS*-codon mutated, or two-or-more-*KRAS*-codons mutated cases. Click here for file

Additional file 2: Table S2 Clinicopathological, and molecular characteristics according to *KRAS* mutation status in 1067 *BRAF*-wild-type cases. Click here for file

Additional file 3: Table S3 Clinicopathological features of 51 *KRAS* codon 61 or 146 mutated cases in 1067 *BRAF*-wild-type cases. Click here for file

Additional file 4: Table S4 Stage I-II, *BRAF*-wild-type colorectal cancer patient mortality according to *KRAS* mutation status. Click here for file

Additional file 5: Table S5 Stage III-IV, *BRAF*-wild-type colorectal cancer patient mortality according to *KRAS* mutation status. Click here for file

Additional file 6: Table S6 Previous studies examining *KRAS* codon 61 and 146 mutations in colorectal cancer. Click here for file

Additional file 7: Figure S1 Pyrosequencing assay design and pyrograms for *KRAS* codons 61 and 146. Click here for file
