# Supplementary material for: Analyses of clinicopathological, molecular, and prognostic associations of KRAS codon 61 and codon 146 mutations in colorectal cancer: cohort study and literature review
Source: Mol Cancer. 2014 May 31;13:135. doi: 10.1186/1476-4598-13-135 (PMC4051153; doi:10.1186/1476-4598-13-135)
Supplement: Additional file 1: Table S1 — Clinicopathological, and molecular characteristics of KRAS-wild-type, only-one-KRAS-codon mutated, or two-or-more-KRAS-codons mutated cases. [file 1476-4598-13-135-S1.doc]

Table S1. Clinicopathological, and molecular characteristics of *KRAS*-wild-type, only-one-*KRAS*-codon mutated, or two-or-more-*KRAS*-codons mutated cases

| Clinicopathological or molecular feature | Total No. | *KRAS*-wild-type | *KRAS* mutant | | | *P*  (*KRAS*-wild-type vs. *KRAS* mutations identified in two or more codons) |
| --- | --- | --- | --- | --- | --- | --- |
|  |  |  | Mutations identified in only one codon | *P*  (Mutations identified in only one codon vs. two or more codons) | Mutations identified in two or more codons |  |
| Total No. of patients | 1267 | 762 | 493 |  | 12 |  |
|  |  |  |  |  |  |  |
| Sex |  |  |  | 0.57 |  | 0.38 |
| Male | 573 (45%) | 322 (42%) | 244 (49%) |  | 7 (58%) |  |
| Female | 694 (55%) | 440 (58%) | 249 (51%) |  | 5 (42%) |  |
|  |  |  |  |  |  |  |
| Mean age (years) ± SD | 68.6 ± 8.7 | 68.4 ± 8.6 | 68.9 ± 8.8 | 0.21 | 65.3 ± 8.8 | 0.27 |
|  |  |  |  |  |  |  |
| BMI (kg/m^2^) |  |  |  | 0.50 |  | 0.48 |
| <30 | 1025 (81%) | 607 (80%) | 407 (83%) |  | 11 (92%) |  |
| ≥30 | 240 (19%) | 155 (20%) | 84 (17%) |  | 1 (8%) |  |
|  |  |  |  |  |  |  |
| Year of diagnosis |  |  |  | 0.039 |  | 0.021 |
| Prior to 1998 | 640 (51%) | 375 (49%) | 255 (52%) |  | 10 (83%) |  |
| 1998 - 2006 | 627 (49%) | 387 (51%) | 238 (48%) |  | 2 (17%) |  |
|  |  |  |  |  |  |  |
| Family history of colorectal  cancer in first degree relative(s) |  |  |  | 0.63 |  | 0.63 |
| Absent | 1026 (81%) | 612 (80%) | 403 (82%) |  | 11 (92%) |  |
| Present in one first degree relative | 179 (14%) | 111 (15%) | 67 (13%) |  | 1 (8%) |  |
| Present in two or more first degree relatives | 62 (5%) | 39 (5%) | 23 (5%) |  | 0 |  |
|  |  |  |  |  |  |  |
| Tumor location |  |  |  | 0.57 |  | 0.44 |
| Cecum | 209 (17%) | 90 (12%) | 116 (24%) |  | 3 (25%) |  |
| Ascending colon | 262 (21%) | 171 (23%) | 87 (18%) |  | 4 (34%) |  |
| Hepatic flexure to 　　　　transverse colon | 117 (9%) | 78 (10%) | 39 (8%) |  | 0 |  |
| Splenic flexure to descending colon | 90 (7%) | 57 (8%) | 32 (7%) |  | 1 (8%) |  |
| Sigmoid colon | 297 (24%) | 182 (24%) | 114 (23%) |  | 1 (8%) |  |
| Rectum | 279 (22%) | 176 (23%) | 100 (20%) |  | 3 (25%) |  |
|  |  |  |  |  |  |  |
| Disease stage |  |  |  | 0.43 |  | 0.18 |
| I | 298 (24%) | 190 (25%) | 105 (21%) |  | 3 (25%) |  |
| II | 354 (28%) | 230 (30%) | 123 (25%) |  | 1 (8.3%) |  |
| III | 328 (26%) | 183 (24%) | 140 (28%) |  | 5 (42%) |  |
| IV | 173 (14%) | 93 (12%) | 77 (16%) |  | 3 (25%) |  |
| Unknown | 114 (9%) | 66 (9%) | 48 (9.7%) |  | 0 |  |
|  |  |  |  |  |  |  |
| Tumor differentiation |  |  |  | 0.51 |  | 0.99 |
| Well-moderate | 1137 (90%) | 663 (88%) | 463 (94%) |  | 11 (92%) |  |
| Poor | 123 (9.8%) | 94 (12%) | 28 (5.7%) |  | 1 (8.3%) |  |
|  |  |  |  |  |  |  |
| Peritumoral lymphocytic reaction |  |  |  | 0.89 |  | 0.66 |
| Absent-minimal | 164 (14%) | 96 (13%) | 67 (14%) |  | 1 (8%) |  |
| Mild | 878 (72%) | 515 (71%) | 353 (75%) |  | 10 (84%) |  |
| Moderate-marked | 170 (14%) | 117 (16%) | 53 (11%) |  | 1 (8%) |  |
|  |  |  |  |  |  |  |
| MSI status |  |  |  | 0.17 |  | 0.99 |
| MSI-low/MSS | 1057 (85%) | 587 (79%) | 460 (94%) |  | 10 (83%) |  |
| MSI-high | 191 (15%) | 160 (21%) | 29 (6%) |  | 2 (17%) |  |
|  |  |  |  |  |  |  |
| CIMP status |  |  |  | 0.14 |  | 0.61 |
| CIMP-negative | 521 (44%) | 311 (44%) | 203 (43%) |  | 7 (58%) |  |
| CIMP-low | 460 (39%) | 224 (32%) | 233 (50%) |  | 3 (25%) |  |
| CIMP-high | 206 (17%) | 172 (24%) | 32 (7%) |  | 2 (17%) |  |
|  |  |  |  |  |  |  |
| *PIK3CA* mutation status |  |  |  | 0.15 |  | 0.0047 |
| Wild-type | 983 (84%) | 632 (89%) | 345 (76%) |  | 6 (55%) |  |
| Mutant | 190 (16%) | 78 (11%) | 107 (24%) |  | 5 (45%) |  |
|  |  |  |  |  |  |  |
| *BRAF* mutation status |  |  |  | 0.16 |  | 0.31 |
| Wild-type | 1078 (85%) | 582 (77%) | 485 (99%) |  | 11 (92%) |  |
| Mutant | 184 (15%) | 177 (23%) | 6 (1%) |  | 1 (8%) |  |
|  |  |  |  |  |  |  |
| Mean LINE-1 methylation level (%) ± SD | 62.7 ± 9.3 | 62.8 ± 9.6 | 62.5 ± 9.0 | 0.17 | 59.5 ± 8.3 | 0.15 |

(%) indicates the proportion of cases with a specific clinicopathological, or molecular feature among each *KRAS* mutation status group.

The *P*-value for significance was adjusted for multiple hypothesis testing to *P*=0.05/14=0.0036. Thus, a *P*-value between 0.05 and 0.0036 should be regarded as of borderline significance.

BMI, body mass index; CIMP, CpG island methylator phenotype; MSI, microsatellite instability; MSS, microsatellite stable; SD, standard deviation
