# Supplementary material for: Analyses of clinicopathological, molecular, and prognostic associations of KRAS codon 61 and codon 146 mutations in colorectal cancer: cohort study and literature review
Source: Mol Cancer. 2014 May 31;13:135. doi: 10.1186/1476-4598-13-135 (PMC4051153; doi:10.1186/1476-4598-13-135)
Supplement: Additional file 2: Table S2 — Clinicopathological, and molecular characteristics according to KRAS mutation status in 1067 BRAF-wild-type cases. [file 1476-4598-13-135-S2.doc]

**Table S2. Clinicopathological, and molecular characteristics according to *KRAS* mutation status in 1067 *BRAF*-wild-type cases**

| Clinicopathological or molecular feature | Total No. | *KRAS* | | *P*  (Wild-type  vs. mutant) | *KRAS* mutations identified in only one codon | | | | *P*  (Across four mutants) |
| --- | --- | --- | --- | --- | --- | --- | --- | --- | --- |
|  |  | Wild-type | Mutant |  | Codon 12 | Codon 13 | Codon 61 | Codon 146 |  |
| Total No. of patients | 1067 | 582 | 485 |  | 328 | 106 | 16 | 35 |  |
|  |  |  |  |  |  |  |  |  |  |
| Sex |  |  |  | 0.49 |  |  |  |  | 0.047 |
| Male | 520 (49%) | 278 (48%) | 242 (50%) |  | 161 (49%) | 59 (56%) | 3 (19%) | 19 (54%) |  |
| Female | 547 (51%) | 304 (52%) | 243 (50%) |  | 167 (51%) | 47 (44%) | 13 (81%) | 16 (46%) |  |
|  |  |  |  |  |  |  |  |  |  |
| Mean age (years) ± SD | 68.4 ± 8.8 | 68.0 ± 8.8 | 68.9 ± 8.8 | 0.11 | 69.6 ± 8.5 | 67.7 ± 9.2 | 69.4 ± 9.2 | 66.0 ± 9.8 | 0.082 |
|  |  |  |  |  |  |  |  |  |  |
| BMI (kg/m^2^) |  |  |  | 0.14 |  |  |  |  | 0.63 |
| <30 | 866 (81%) | 464 (80%) | 402 (83%) |  | 275 (84%) | 86 (81%) | 11 (73%) | 30 (86%) |  |
| ≥30 | 199 (19%) | 118 (20%) | 81 (17%) |  | 52 (16%) | 20 (19%) | 4 (27%) | 5 (14%) |  |
|  |  |  |  |  |  |  |  |  |  |
| Year of diagnosis |  |  |  | 0.85 |  |  |  |  | 0.042 |
| Prior to 1998 | 549 (51%) | 301 (52%) | 248 (51%) |  | 159 (48%) | 61 (58%) | 5 (31%) | 23 (66%) |  |
| 1998 - 2006 | 518 (49%) | 281 (48%) | 237 (49%) |  | 169 (52%) | 45 (42%) | 11 (69%) | 12 (34%) |  |
|  |  |  |  |  |  |  |  |  |  |
| Family history of colorectal  cancer in first degree relative(s) |  |  |  | 0.90 |  |  |  |  | 0.87 |
| Absent | 863 (81%) | 468 (80%) | 395 (81%) |  | 268 (82%) | 87 (82%) | 13 (81%) | 27 (77%) |  |
| Present in one first degree relative | 153 (14%) | 86 (15%) | 67 (14%) |  | 44 (13%) | 15 (14%) | 3 (19%) | 5 (14%) |  |
| Present in two or more first degree relatives | 51 (5%) | 28 (5%) | 23 (5%) |  | 16 (5%) | 4 (4%) | 0 | 3 (9%) |  |
|  |  |  |  |  |  |  |  |  |  |
| Tumor location |  |  |  | <0.0001 |  |  |  |  | 0.81 |
| Cecum | 181 (17%) | 65 (11%) | 116 (24%) |  | 79 (24%) | 27 (25%) | 4 (25%) | 6 (17%) |  |
| Ascending colon | 164 (16%) | 79 (14%) | 85 (18%) |  | 52 (16%) | 23 (21%) | 3 (19%) | 7 (20%) |  |
| Hepatic flexure to transverse colon | 86 (8%) | 48 (8%) | 38 (8%) |  | 26 (8%) | 7 (7%) | 3 (19%) | 2 (6%) |  |
| Splenic flexure to descending colon | 75 (7%) | 43 (7%) | 32 (7%) |  | 22 (7%) | 7 (7%) | 0 | 3 (9%) |  |
| Sigmoid colon | 282 (27%) | 171 (30%) | 111 (23%) |  | 80 (25%) | 22 (21%) | 1 (6%) | 8 (24%) |  |
| Rectum | 268 (25%) | 170 (30%) | 98 (20%) |  | 65 (20%) | 20 (19%) | 5 (31%) | 8 (24%) |  |
|  |  |  |  |  |  |  |  |  |  |
| Disease stage |  |  |  | 0.0048 |  |  |  |  | 0.91 |
| I | 263 (25%) | 159 (27%) | 104 (21%) |  | 76 (23%) | 20 (19%) | 4 (25%) | 4 (11%) |  |
| II | 281 (26%) | 163 (28%) | 118 (24%) |  | 75 (23%) | 28 (26%) | 4 (25%) | 11 (32%) |  |
| III | 281 (26%) | 143 (25%) | 138 (29%) |  | 95 (29%) | 29 (27%) | 3 (19%) | 11 (32%) |  |
| IV | 133 (13%) | 56 (9%) | 77 (16%) |  | 51 (16%) | 18 (17%) | 2 (12%) | 6 (17%) |  |
| Unknown | 109 (10%) | 61 (11%) | 48 (10%) |  | 31 (9%) | 11 (11%) | 3 (19%) | 3 (8%) |  |
|  |  |  |  |  |  |  |  |  |  |
| Tumor differentiation |  |  |  | 0.32 |  |  |  |  | 0.71 |
| Well to moderate | 992 (94%) | 536 (93%) | 456 (94%) |  | 309 (95%) | 98 (92%) | 15 (94%) | 34 (97%) |  |
| Poor | 68 (6%) | 41 (7%) | 27 (6%) |  | 17 (5%) | 8 (8%) | 1 (6%) | 1 (3%) |  |
|  |  |  |  |  |  |  |  |  |  |
| Peritumoral lymphocytic reaction |  |  |  | 0.42 |  |  |  |  | 0.51 |
| Absent/minimal | 141 (14%) | 74 (13%) | 67 (14%) |  | 47 (15%) | 14 (13%) | 2 (13%) | 4 (12%) |  |
| Mild | 755 (74%) | 407 (74%) | 348 (75%) |  | 234 (75%) | 75 (72%) | 11 (74%) | 28 (85%) |  |
| Moderate/marked | 122 (12%) | 73 (13%) | 49 (11%) |  | 30 (10%) | 16 (15%) | 2 (13%) | 1 (3%) |  |
|  |  |  |  |  |  |  |  |  |  |
| MSI status |  |  |  | 0.0022 |  |  |  |  | 0.042 |
| MSI-low/MSS | 961 (92%) | 507 (89%) | 454 (94%) |  | 311 (95%) | 99 (95%) | 13 (81%) | 31 (89%) |  |
| MSI-high | 89 (8%) | 62 (11%) | 27 (6%) |  | 15 (5%) | 5 (5%) | 3 (19%) | 4 (11%) |  |
|  |  |  |  |  |  |  |  |  |  |
| CIMP status |  |  |  | <0.0001 |  |  |  |  | 0.0072 |
| CIMP-negative | 499 (50%) | 300 (56%) | 199 (43%) |  | 135 (44%) | 37 (37%) | 8 (53%) | 19 (54%) |  |
| CIMP-low | 419 (42%) | 189 (35%) | 230 (50%) |  | 153 (50%) | 58 (57%) | 3 (20%) | 16 (46%) |  |
| CIMP-high | 77 (8%) | 46 (9%) | 31 (7%) |  | 21 (6%) | 6 (6%) | 4 (27%) | 0 |  |
|  |  |  |  |  |  |  |  |  |  |
| *PIK3CA* mutation status |  |  |  | <0.0001 |  |  |  |  | 0.68 |
| Wild-type | 829 (84%) | 491 (91%) | 338 (76%) |  | 237 (77%) | 71 (74%) | 11 (79%) | 19 (68%) |  |
| Mutant | 157 (16%) | 50 (9%) | 107 (24%) |  | 70 (23%) | 25 (26%) | 3 (21%) | 9 (32%) |  |
|  |  |  |  |  |  |  |  |  |  |
| Mean LINE-1 methylation level (%) ± SD | 62.1 ± 9.1 | 61.7 ± 9.2 | 62.6 ± 8.9 | 0.17 | 62.9 ± 9.1 | 61.4 ± 8.2 | 64.5 ± 10.4 | 63.1 ± 9.0 | 0.27 |

(%) indicates the proportion of cases with a specific clinicopathological, or molecular feature among each *KRAS* mutation status group. The *P*-value for significance was adjusted for multiple hypothesis testing to *P*=0.05/14=0.0036. Thus, a *P*-value between 0.05 and 0.0036 should be regarded as of borderline significance. BMI, body mass index; CIMP, CpG island methylator phenotype; MSI, microsatellite instability; MSS, microsatellite stable; SD, standard deviation
