# Supplementary material for: Analyses of clinicopathological, molecular, and prognostic associations of KRAS codon 61 and codon 146 mutations in colorectal cancer: cohort study and literature review
Source: Mol Cancer. 2014 May 31;13:135. doi: 10.1186/1476-4598-13-135 (PMC4051153; doi:10.1186/1476-4598-13-135)
Supplement: Additional file 3: Table S3 — Clinicopathological features of 51 KRAS codon 61 or 146 mutated cases in 1067 BRAF-wild-type cases. [file 1476-4598-13-135-S3.doc]

Table S3. Clinicopathological features of 51 *KRAS* codon 61 or 146 mutated cases in 1067 *BRAF*-wild-type cases

| Patient | *KRAS* | | Sex | Age | BMI (kg/m^2^) | Year of diagnosis | Family history of CRC in first degree of relative(s) | Tumor location | Disease stage | Tumor differentiation | Peritumoral lymphocytic reaction | MSI status | CIMP status | *PIK3CA* mutation | LINE-1 methylation level (%) | Survival status | Follow-up time (year) |
| --- | --- | --- | --- | --- | --- | --- | --- | --- | --- | --- | --- | --- | --- | --- | --- | --- | --- |
|  | Nucleotide change | Amino acid change |  |  |  |  |  |  |  |  |  |  |  |  |  |  |  |
| 1 | c.182A>G | p.Q61R | M | 68.9 | 31.7 | 2000 | Abscent | Rectum | I | Poor | Absent-minimal | MSS | Negative | MUT | 78.2 | Alive | 11.1 |
| 2 | c.182A>T | p.Q61L | F | 53.3 | 27.5 | 1997 | Abscent | Cecum | III | Well-moderate | Mild | MSS | Negative | WT | 57.2 | CSD | 2.4 |
| 3 | c.182A>T | p.Q61L | F | 67.1 | 28.2 | 1999 | Abscent | Ascending | III | Well-moderate | Mild | MSS | Negative | — | 65.1 | Alive | 11.7 |
| 4 | c.182A>T | p.Q61L | F | 78.4 | 30.7 | 2003 | Abscent | Cecum | II | Well-moderate | Mild | MSS | Low | WT | 46.2 | Alive | 7.3 |
| 5 | c.182A>T | p.Q61L | M | 80.2 | 23.5 | 2008 | Abscent | Hepatic flexure | U | Well-moderate | — | MSS | Negative | WT | — | OTH | 0.7 |
| 6 | c.183A>C | p.Q61H | F | 52.9 | 30.8 | 1992 | Abscent | Rectum | U | Well-moderate | Mild | MSS | Low | WT | 70.4 | Alive | 18.4 |
| 7 | c.183A>C | p.Q61H | F | 66.7 | 26.6 | 1994 | Abscent | Sigmoid | III | Well-moderate | Mild | MSS | Negative | — | 67.7 | OTH | 10.1 |
| 8 | c.183A>C | p.Q61H | F | 68.9 | 25.9 | 1994 | Abscent | Rectum | U | Well-moderate | Mild | MSS | Negative | WT | 64.7 | CSD | 6.2 |
| 9 | c.183A>C | p.Q61H | F | 65.3 | 18.6 | 1997 | 1+ | Transverse | II | Well-moderate | Mild | MSS | Negative | WT | 51.6 | OTH | 8.8 |
| 10 | c.183A>C | p.Q61H | F | 75.8 | 28.4 | 2001 | Abscent | Ascending | I | Well-moderate | Mild | MSS | Negative | WT | 56.6 | Alive | 9.9 |
| 11 | c.183A>C | p.Q61H | M | 61.6 | 25.9 | 2002 | Abscent | Cecum | IV | Well-moderate | Mild | MSS | — | WT | 49.3 | Alive | 8.7 |
| 12 | c.183A>C | p.Q61H | F | 65.4 | — | 2002 | Abscent | Rectum | IV | Well-moderate | Moderate-marked | MSS | Low | MUT | 67.9 | CSD | 2.8 |
| 13 | c.180_181delinsAA | p.Q61K | F | 71.8 | 28.6 | 2004 | Abscent | Rectum | II | Well-moderate | Mild | MSI-high | High | WT | 65.2 | CSD | 1.3 |
| 14 | c.180_181delinsAA | p.Q61K | F | 67.8 | 31.1 | 2006 | Abscent | Transverse | I | Well-moderate | Moderate-marked | MSI-high | High | MUT | 78.1 | OTH | 2.7 |
| 15 | c.180_181delinsAA | p.Q61K | F | 80.7 | 18.8 | 2006 | 1+ | Ascending | I | Well-moderate | Mild | MSI-high | High | WT | 70.7 | Alive | 4.6 |
| 16 | c.180_181delinsAA | p.Q61K | F | 85.8 | 24.2 | 2007 | 1+ | Cecum | II | Well-moderate | Absent-minimal | MSS | High | WT | 78.5 | Alive | 3.1 |
| 17 | c.436G>A | p.A146T | F | 54.3 | 22.0 | 1982 | 1+ | Splenic flexure | I | Well-moderate | Mild | MSS | Negative | — | 55.1 | Alive | 28.9 |
| 18 | c.436G>A | p.A146T | F | 49.1 | 22.4 | 1989 | Abscent | Sigmoid | I | Well-moderate | Mild | MSS | Negative | WT | 63.1 | Alive | 21.7 |
| 19 | c.436G>A | p.A146T | M | 59.8 | 28.5 | 1992 | Abscent | Sigmoid | III | Well-moderate | Mild | MSS | Low | WT | 47.2 | Alive | 18.8 |
| 20 | c.436G>A | p.A146T | M | 63.9 | 23.6 | 1992 | 1+ | Cecum | II | Well-moderate | Mild | MSI-low | Negative | WT | 45.0 | Alive | 19.1 |
| 21 | c.436G>A | p.A146T | M | 61.1 | 25.9 | 1992 | Abscent | Cecum | II | Well-moderate | Mild | MSS | Negative | WT | 62.2 | Alive | 19.2 |
| 22 | c.436G>A | p.A146T | M | 67.1 | 26.0 | 1992 | 1+ | Sigmoid | II | Well-moderate | Mild | MSS | Low | WT | 57.4 | CSD | 1.6 |
| 23 | c.436G>A | p.A146T | F | 62.1 | 22.7 | 1992 | Abscent | Sigmoid | III | Well-moderate | Mild | MSS | Low | WT | 67.5 | Alive | 18.8 |
| 24 | c.436G>A | p.A146T | M | 77.8 | 28.4 | 1995 | 1+ | Sigmoid | II | Well-moderate | Mild | MSS | Negative | — | 69.0 | OTH | 9.0 |
| 25 | c.436G>A | p.A146T | F | 56.4 | 26.7 | 1995 | Abscent | Rectum | III | Well-moderate | Mild | MSS | Negative | WT | 58.8 | CSD | 1.9 |
| 26 | c.436G>A | p.A146T | M | 67.2 | 34.0 | 1996 | Abscent | Ascending | II | Well-moderate | Mild | MSI-high | Low | WT | 76.8 | Alive | 14.8 |
| 27 | c.436G>A | p.A146T | F | 66.1 | 27.5 | 1996 | Abscent | Rectum | IV | Well-moderate | Mild | MSS | Negative | WT | 54.6 | CSD | 0.2 |
| 28 | c.436G>A | p.A146T | M | 85.8 | 29.4 | 1997 | 1+ | Cecum | II | Well-moderate | Mild | MSS | Negative | MUT | 62.7 | Alive | 14.0 |
| 29 | c.436G>A | p.A146T | M | 67.2 | 27.6 | 1997 | Abscent | Transverse | II | Well-moderate | Mild | MSS | Negative | — | 61.7 | Alive | 13.3 |
| 30 | c.436G>A | p.A146T | F | 55.4 | 40.0 | 1997 | Abscent | Ascending | III | Well-moderate | Mild | MSS | Negative | — | 49.6 | Alive | 13.9 |
| 31 | c.436G>A | p.A146T | F | 56.8 | 23.1 | 1998 | Abscent | Rectum | IV | Well-moderate | Mild | MSS | Negative | WT | 68.8 | Alive | 12.6 |
| 32 | c.436G>A | p.A146T | M | 66.5 | 27.4 | 1999 | Abscent | Sigmoid | II | Well-moderate | Absent-minimal | MSS | Negative | WT | 74.7 | Alive | 11.6 |
| 33 | c.436G>A | p.A146T | M | 74.4 | 30.4 | 1999 | ≥2+ | Ascending | II | Well-moderate | Mild | MSI-high | Low | MUT | 82.6 | OTH | 1.8 |
| 34 | c.436G>A | p.A146T | M | 78.3 | 28.6 | 2001 | ≥2+ | Splenic flexure | IV | Well-moderate | Moderate-marked | MSI-high | Low | MUT | 66.9 | Alive | 9.5 |
| 35 | c.436G>A | p.A146T | F | 80.3 | 22.9 | 2002 | Abscent | Cecum | III | Well-moderate | Mild | MSS | Negative | MUT | 54.1 | Alive | 8.3 |
| 36 | c.436G>A | p.A146T | F | 78.8 | 31.7 | 2002 | Abscent | Sigmoid | U | Well-moderate | Mild | MSS | Low | WT | 76.8 | Alive | 8.9 |
| 37 | c.436G>A | p.A146T | M | 73.4 | 28.1 | 2007 | ≥2+ | Ascending | III | Well-moderate | Mild | MSS | Low | MUT | 69.0 | Alive | 3.9 |
| 38 | c.436G>C | p.A146P | F | 55.3 | 24.0 | 1997 | Abscent | Rectum | I | Well-moderate | Mild | MSS | Negative | MUT | 57.9 | Alive | 13.8 |
| 39 | c.436G>C | p.A146P | M | 81.8 | 27.3 | 1998 | Abscent | — | IV | Well-moderate | Absent-minimal | MSS | Negative | WT | 47.8 | CSD | 1.1 |
| 40 | c.436G>C | p.A146P | M | 70.1 | 25.9 | 2008 | Abscent | Rectum | I | Well-moderate | — | MSS | Negative | WT | 69.3 | Alive | 3.2 |
| 41 | c.437C>T | p.A146V | F | 56.2 | 26.6 | 1984 | Abscent | Rectum | III | Well-moderate | Mild | MSS | Low | MUT | 69.7 | CSD | 1.7 |
| 42 | c.437C>T | p.A146V | F | 54.3 | 23.2 | 1987 | Abscent | Ascending | IV | Well-moderate | Mild | MSS | Low | MUT | 62.5 | CSD | 0.9 |
| 43 | c.437C>T | p.A146V | M | 63.1 | 27.5 | 1991 | Abscent | Ascending | III | Poor | Mild | MSS | Negative | — | 56.0 | CSD | 1.1 |
| 44 | c.437C>T | p.A146V | M | 74.8 | 28.7 | 1992 | Abscent | Ascending | III | Well-moderate | Mild | MSS | Low | WT | 63.4 | OTH | 12.1 |
| 45 | c.437C>T | p.A146V | M | 76.3 | 26.8 | 1993 | Abscent | Cecum | IV | Well-moderate | Mild | MSS | Low | WT | 63.2 | CSD | 0.5 |
| 46 | c.437C>T | p.A146V | F | 57.8 | 29.2 | 1993 | Abscent | Rectum | U | Well-moderate | — | MSS | Low | — | 73.7 | OTH | 13.0 |
| 47 | c.437C>T | p.A146V | M | 54.3 | 31.6 | 1995 | Abscent | Cecum | II | Well-moderate | Mild | MSI-high | Low | — | 70.4 | Alive | 16.1 |
| 48 | c.437C>T | p.A146V | M | 63.5 | 25.5 | 1995 | Abscent | Sigmoid | U | Well-moderate | Mild | MSS | Low | WT | 63.1 | OTH | 1.4 |
| 49 | c.437C>T | p.A146V | F | 55.0 | 27.7 | 1999 | Abscent | Transverse | III | Well-moderate | Mild | MSS | Negative | WT | 62.3 | Alive | 11.9 |
| 50 | c.437C>T | p.A146V | F | 77.3 | 27.0 | 2002 | Abscent | Rectum | II | Well-moderate | Absent-minimal | MSS | Negative | MUT | 55.3 | Alive | 8.3 |
| 51 | c.437C>T | p.A146V | F | 69.1 | 27.7 | 2008 | Abscent | Descending | III | Well-moderate | Absent-minimal | MSS | Low | WT | 72.0 | Alive | 2.8 |

BMI, body mass index; CIMP, CpG island methylator phenotype; CSD, colorectal cancer-specific death; CRC, colorectal cancer;

MSI, microsatellite instability; MSS, microsatellite stable; MUT, mutant; OTH, death from other causes than colorectal cancer;

U, Unknown; WT, wild-type; 1+, present in one first degree of relative; ≥2+, present in two or more first degree of relatives
