# Supplementary material for: Analyses of clinicopathological, molecular, and prognostic associations of KRAS codon 61 and codon 146 mutations in colorectal cancer: cohort study and literature review
Source: Mol Cancer. 2014 May 31;13:135. doi: 10.1186/1476-4598-13-135 (PMC4051153; doi:10.1186/1476-4598-13-135)
Supplement: Additional file 4: Table S4 — Stage I-II, BRAF-wild-type colorectal cancer patient mortality according to KRAS mutation status. [file 1476-4598-13-135-S4.doc]

Table S4. Stage I-II, *BRAF*-wild-type colorectal cancer patient mortality according to *KRAS* mutation status

| *KRAS* | Total No. | Colorectal cancer-specific mortality | | | |  | Overall mortality | | |
| --- | --- | --- | --- | --- | --- | --- | --- | --- | --- |
|  |  | No. of events | | Univariate  HR  (95% CI) | Multivariate stage-stratified  HR  (95% CI) |  | No. of events | Univariate  HR  (95% CI) | Multivariate stage-stratified  HR  (95% CI) |
| Wild-type (codons 12, 13, 61 and 146) | 322 | 29 | | 1 (referent) | 1 (referent) |  | 104 | 1 (referent) | 1 (referent) |
|  |  |  | |  |  |  |  |  |  |
| All mutants together | 222 | 36 | | 1.93 (1.18-3.15) | 2.30 (1.37-3.87) |  | 91 | 1.40 (1.06-1.86) | 1.44 (1.06-1.97) |
|  |  |  | | *P*=0.0085 | *P*=0.0017 |  |  | *P*=0.019 | *P*=0.022 |
|  |  |  | |  |  |  |  |  |  |
| Codons 12 and 13, and codons 61 and 146 |  |  | |  |  |  |  |  |  |
| Codons 12 and 13 | 199 | 34 | | 2.03 (1.23-3.33) | 2.45 (1.47-4.16) |  | 85 | 1.46 (1.10-1.95) | 1.48 (1.08-2.04) |
|  |  |  | | *P*=0.0053 | *P*=0.0009 |  |  | *P*=0.0093 | *P*=0.016 |
|  |  |  | |  |  |  |  |  |  |
| Codons 61 and 146 | 23 | 2 | | 1.07 (0.26-4.50) | 1.11 (0.25-4.91) |  | 6 | 0.87 (0.38-1.99) | 1.08 (0.46-2.51) |
|  |  |  | |  |  |  |  |  |  |
|  |  |  | |  |  |  |  |  |  |
| Codons 12, 13, 61 and 146 |  |  | |  |  |  |  |  |  |
| Codon 12 mutants | 151 | 28 | | 2.25 (1.34-3.78) | 2.62 (1.52-4.52) |  | 68 | 1.61 (1.18-2.18) | 1.60 (1.14-2.23) |
|  |  |  | | *P*=0.0023 | *P*=0.0005 |  |  | *P*=0.0025 | *P*=0.0059 |
|  |  |  | |  |  |  |  |  |  |
| Codon 13 mutants | 48 | 6 | | 1.39 (0.58-3.35) | 1.82 (0.73-4.54) |  | 17 | 1.09 (0.65-1.82) | 1.11 (0.64-1.91) |
|  |  |  | |  |  |  |  |  |  |
|  |  |  | |  |  |  |  |  |  |
| Codon 61 mutants | 8 | 1 | | 2.08 (0.28-15.3) | 1.65 (0.19-14.4) |  | 3 | 2.38 (0.75-7.53) | 1.97 (0.60-6.46) |
|  |  |  | |  |  |  |  |  |  |
|  |  |  | |  |  |  |  |  |  |
| Codon 146 mutants | 15 | 1 | | 0.72 (0.10-5.30) | 0.86 (0.12-6.48) |  | 3 | 0.53 (0.17-1.68) | 0.72 (0.22-2.35) |
|  |  |  | |  |  |  |  |  |  |
|  |  |  | |  |  |  |  |  |  |
| The 10 most common mutations in codons 12, 13, 61 and 146 | | | | |  |  |  |  |  |
| c.34G>A (p.G12S) | 5 | 1 | 2.11 (0.29-15.5) | | 2.46 (0.33-18.4) |  | 1 | 0.51 (0.07-3.65) | 0.82 (0.11-5.99) |
|  |  |  |  | |  |  |  |  |  |
|  |  |  |  | |  |  |  |  |  |
| c.34G>C (p.G12R) | 3 | 1 | 5.22 (0.71-38.6) | | 4.74 (0.60-37.8) |  | 2 | 4.19 (1.02-17.2) | 5.37 (1.24-23.2) |
|  |  |  |  | |  |  |  | *P*=0.046 | *P*=0.025 |
|  |  |  |  | |  |  |  |  |  |
| c.34G>T (p.G12C) | 23 | 6 | 3.29 (1.36-7.92) | | 3.89 (1.55-9.75) |  | 13 | 2.12 (1.19-3.79) | 2.41 (1.32-4.39) |
|  |  |  | *P*=0.0081 | | *P*=0.0037 |  |  | *P*=0.011 | *P*=0.0042 |
|  |  |  |  | |  |  |  |  |  |
| c.35G>A (p.G12D) | 73 | 11 | 1.86 (0.93-3.73) | | 2.43 (1.18-5.01) |  | 29 | 1.47 (0.97-2.22) | 1.44 (0.92-2.23) |
|  |  |  |  | | *P*=0.017 |  |  |  |  |
|  |  |  |  | |  |  |  |  |  |
| c.35G>C (p.G12A) | 5 | 1 | 2.10 (0.29-15.4) | | 2.42 (0.32-18.4) |  | 2 | 1.10 (0.27-4.47) | 0.77 (0.19-3.22) |
|  |  |  |  | |  |  |  |  |  |
|  |  |  |  | |  |  |  |  |  |
| c.35G>T (p.G12V) | 42 | 8 | 2.24 (1.02-4.90) | | 2.20 (0.99-4.89) |  | 21 | 1.72 (1.07-2.75) | 1.57 (0.96-2.56) |
|  |  |  | *P*=0.044 | |  |  |  | *P*=0.025 |  |
|  |  |  |  | |  |  |  |  |  |
| c.38G>A (p.G13D) | 45 | 6 | 1.50 (0.62-3.61) | | 1.94 (0.77-4.87) |  | 16 | 1.10 (0.65-1.87) | 1.07 (0.61-1.88) |
|  |  |  |  | |  |  |  |  |  |
|  |  |  |  | |  |  |  |  |  |
| c.183A>C (p.Q61H) | 2 | 0 | - | | - |  | 1 | 1.88 (0.26-13.6) | 1.99 (0.27-14.6) |
|  |  |  |  | |  |  |  |  |  |
|  |  |  |  | |  |  |  |  |  |
| c.436G>A (p.A146T) | 11 | 1 | 0.96 (0.13-7.08) | | 1.21 (0.16-9.28) |  | 3 | 0.68 (0.22-2.16) | 0.80 (0.24-2.62) |
|  |  |  |  | |  |  |  |  |  |
|  |  |  |  | |  |  |  |  |  |
| c.437C>T (p.A146V) | 2 | 0 | - | | - |  | 0 | - | - |

The multivariate Cox regression model included the same set of covariates selected as in Table 3.

For the survival analysis of mutations in the two groups of *KRAS* codons (codons 12 and 13, and codons 61 and 146), the *P*-value for significance was adjusted for multiple hypothesis testing to *P*=0.05/2=0.025. Thus, a *P*-value between 0.05 and 0.025 should be regarded as of borderline significance.

For the survival analysis of mutations in the four *KRAS* codons (12, 13, 61 and 146), the *P*-value for significance was adjusted for multiple hypothesis testing to *P*=0.05/4=0.013. Thus, a *P*-value between 0.05 and 0.013 should be regarded as of borderline significance.

For the survival analysis of the 10 most common *KRAS* mutations, the *P*-value for significance was adjusted for multiple hypothesis testing to *P*=0.05/10=0.005. Thus, a *P*-value between 0.05 and 0.005 should be regarded as of borderline significance.

CI, confidence interval; HR, hazard ratio
