# Supplementary material for: Analyses of clinicopathological, molecular, and prognostic associations of KRAS codon 61 and codon 146 mutations in colorectal cancer: cohort study and literature review
Source: Mol Cancer. 2014 May 31;13:135. doi: 10.1186/1476-4598-13-135 (PMC4051153; doi:10.1186/1476-4598-13-135)
Supplement: Additional file 5: Table S5 — Stage III-IV, BRAF-wild-type colorectal cancer patient mortality according to KRAS mutation status. [file 1476-4598-13-135-S5.doc]

Table S5. Stage III-IV, *BRAF*-wild-type colorectal cancer patient mortality according to *KRAS* mutation status

| *KRAS* | Total No. | Colorectal cancer-specific mortality | | | |  | Overall mortality | | |
| --- | --- | --- | --- | --- | --- | --- | --- | --- | --- |
|  |  | No. of events | | Univariate  HR  (95% CI) | Multivariate stage-stratified  HR  (95% CI) |  | No. of events | Univariate  HR  (95% CI) | Multivariate stage-stratified  HR  (95% CI) |
| Wild-type (codons 12, 13, 61 and 146) | 199 | 96 | | 1 (referent) | 1 (referent) |  | 122 | 1 (referent) | 1 (referent) |
|  |  |  | |  |  |  |  |  |  |
| All mutants together | 215 | 120 | | 1.26 (0.96-1.65) | 1.19 (0.89-1.59) |  | 138 | 1.15 (0.90-1.46) | 1.10 (0.84-1.43) |
|  |  |  | |  |  |  |  |  |  |
|  |  |  | |  |  |  |  |  |  |
| Codons 12 and 13, and codons 61 and 146 |  |  | |  |  |  |  |  |  |
| Codons 12 and 13 | 193 | 111 | | 1.31 (0.99-1.72) | 1.22 (0.91-1.64) |  | 127 | 1.19 (0.92-1.52) | 1.10 (0.83-1.44) |
|  |  |  | |  |  |  |  |  |  |
|  |  |  | |  |  |  |  |  |  |
| Codons 61 and 146 | 22 | 9 | | 0.86 (0.43-1.70) | 0.89 (0.44-1.81) |  | 11 | 0.83 (0.44-1.54) | 1.10 (0.58-2.09) |
|  |  |  | |  |  |  |  |  |  |
|  |  |  | |  |  |  |  |  |  |
| Codons 12, 13, 61 and 146 |  |  | |  |  |  |  |  |  |
| Codon 12 mutants | 146 | 87 | | 1.40 (1.04-1.87) | 1.37 (1.00-1.88) |  | 99 | 1.27 (0.97-1.66) | 1.20 (0.90-1.59) |
|  |  |  | | *P*=0.026 | *P*=0.049 |  |  |  |  |
|  |  |  | |  |  |  |  |  |  |
| Codon 13 mutants | 47 | 24 | | 1.07 (0.68-1.68) | 0.84 (0.52-1.36) |  | 28 | 0.96 (0.64-1.46) | 0.82 (0.52-1.28) |
|  |  |  | |  |  |  |  |  |  |
|  |  |  | |  |  |  |  |  |  |
| Codon 61 mutants | 5 | 2 | | 0.72 (0.18-2.93) | 0.63 (0.15-2.66) |  | 3 | 0.89 (0.28-2.81) | 1.28 (0.39-4.20) |
|  |  |  | |  |  |  |  |  |  |
|  |  |  | |  |  |  |  |  |  |
| Codon 146 mutants | 17 | 7 | | 0.91 (0.42-1.96) | 1.00 (0.46-2.21) |  | 8 | 0.81 (0.39-1.65) | 1.03 (0.49-2.16) |
|  |  |  | |  |  |  |  |  |  |
|  |  |  | |  |  |  |  |  |  |
| The 10 most common mutations in codons 12, 13, 61 and 146 | | | | |  |  |  |  |  |
| c.34G>A (p.G12S) | 6 | 5 | 2.73 (1.09-6.80) | | 0.92 (0.35-2.42) |  | 5 | 2.36 (0.95-5.85) | 0.80 (0.31-2.09) |
|  |  |  | *P*=0.031 | |  |  |  |  |  |
|  |  |  |  | |  |  |  |  |  |
| c.34G>C (p.G12R) | 4 | 4 | 7.17 (2.50-20.6) | | 2.75 (0.86-8.79) |  | 4 | 6.75 (2.35-19.3) | 2.02 (0.64-6.34) |
|  |  |  | *P*=0.0003 | |  |  |  | *P*=0.0004 |  |
|  |  |  |  | |  |  |  |  |  |
| c.34G>T (p.G12C) | 17 | 10 | 1.41 (0.73-2.72) | | 2.49 (1.24-5.00) |  | 11 | 1.23 (0.66-2.29) | 1.41 (0.72-2.75) |
|  |  |  |  | | *P*=0.010 |  |  |  |  |
|  |  |  |  | |  |  |  |  |  |
| c.35G>A (p.G12D) | 67 | 38 | 1.31 (0.90-1.92) | | 1.12 (0.74-1.69) |  | 45 | 1.27 (0.90-1.79) | 1.09 (0.75-1.57) |
|  |  |  |  | |  |  |  |  |  |
|  |  |  |  | |  |  |  |  |  |
| c.35G>C (p.G12A) | 11 | 5 | 0.89 (0.36-2.19) | | 0.55 (0.22-1.41) |  | 6 | 0.76 (0.34-1.74) | 0.55 (0.23-1.27) |
|  |  |  |  | |  |  |  |  |  |
|  |  |  |  | |  |  |  |  |  |
| c.35G>T (p.G12V) | 41 | 25 | 1.38 (0.88-2.15) | | 2.38 (1.49-3.80) |  | 28 | 1.23 (0.81-1.86) | 2.00 (1.28-3.12) |
|  |  |  |  | | *P*=0.0003 |  |  |  | *P*=0.0022 |
|  |  |  |  | |  |  |  |  |  |
| c.38G>A (p.G13D) | 45 | 24 | 1.14 (0.73-1.79) | | 0.85 (0.53-1.38) |  | 27 | 1.00 (0.66-1.53) | 0.83 (0.53-1.31) |
|  |  |  |  | |  |  |  |  |  |
|  |  |  |  | |  |  |  |  |  |
| c.183A>C (p.Q61H) | 3 | 1 | 0.57 (0.08-4.14) | | 0.32 (0.04-2.41) |  | 2 | 0.96 (0.24-3.91) | 1.01 (0.24-4.29) |
|  |  |  |  | |  |  |  |  |  |
|  |  |  |  | |  |  |  |  |  |
| c.436G>A (p.A146T) | 9 | 2 | 0.40 (0.10-1.61) | | 0.43 (0.10-1.79) |  | 2 | 0.30 (0.07-1.21) | 0.44 (0.11-1.85) |
|  |  |  |  | |  |  |  |  |  |
|  |  |  |  | |  |  |  |  |  |
| c.437C>T (p.A146V) | 7 | 4 | 1.61 (0.59-4.41) | | 2.44 (0.86-6.94) |  | 5 | 1.66 (0.67-4.09) | 2.36 (0.92-6.08) |

The multivariate Cox regression model included the same set of covariates selected as in Table 3.

For the survival analysis of mutations in the two groups of *KRAS* codons (codons 12 and 13, and codons 61 and 146), the *P*-value for significance was adjusted for multiple hypothesis testing to *P*=0.05/2=0.025. Thus, a *P*-value between 0.05 and 0.025 should be regarded as of borderline significance.

For the survival analysis of mutations in the four *KRAS* codons (12, 13, 61 and 146), the *P*-value for significance was adjusted for multiple hypothesis testing to *P*=0.05/4=0.013. Thus, a *P*-value between 0.05 and 0.013 should be regarded as of borderline significance.

For the survival analysis of the 10 most common *KRAS* mutations, the *P*-value for significance was adjusted for multiple hypothesis testing to *P*=0.05/10=0.005. Thus, a *P*-value between 0.05 and 0.005 should be regarded as of borderline significance.

CI, confidence interval; HR, hazard ratio
