# Supplementary material for: Analyses of clinicopathological, molecular, and prognostic associations of KRAS codon 61 and codon 146 mutations in colorectal cancer: cohort study and literature review
Source: Mol Cancer. 2014 May 31;13:135. doi: 10.1186/1476-4598-13-135 (PMC4051153; doi:10.1186/1476-4598-13-135)
Supplement: Additional file 6: Table S6 — Previous studies examining KRAS codon 61 and 146 mutations in colorectal cancer. [file 1476-4598-13-135-S6.doc]

Table S6. Previous studies examining *KRAS* codon 61 and 146 mutations in colorectal cancer

| Reference | No. of hospitals | Tumor location | Disease stage/  No. of cases | Sequencing method | *KRAS* mutant | | | | | | | | | | | | | | | |
| --- | --- | --- | --- | --- | --- | --- | --- | --- | --- | --- | --- | --- | --- | --- | --- | --- | --- | --- | --- | --- |
|  |  |  |  |  | Codon 61 | | | | | | | | | | |  | Codon 146 | | | |
|  |  |  |  |  | c.180_181delinsAA (p.Q61K) | c.181  C>A (p.Q61K) | | | c.181  C>G (p.Q61E) | c.182  A>G (p.Q61R) | c.182  A>T (p.Q61L) | | c.183  A>C (p.Q61H) | | c.183  A>T (p.Q61H) |  | c.436  G>A (p.A146T) | c.436  G>C (p.A146P) | | c.437  C>T (p.A146V) |
| Current  study | M | CR | I-IV/  1267 | Pyro-  sequencing | 5 (0.4%) | — | | | — | 2 (0.2%) | 4 (0.3%) | | 7 (0.6%) | | 1 (0.1%) |  | 25 (2.0%) | 3 (0.2%) | | 12 (0.9%) |
|  |  |  |  |  | [19 (1.5%)] | | | | | | | | | | |  | [40 (3.2%)] | | | |
| 32 | 2 | CR | NA/  1506 | Sanger | — | | 1 (0.1%) | — | | 2 (0.1%) | | 5 (0.3%) | | 9 (0.6%) | |  | 18 (1.2%) | | — | — |
|  |  |  |  |  | [18 (1.2%)] | | | | | | | | | | |  |  |  |  |  |
| 27 | M | CR | IV/  747 | MALDI-TOF /MassARRAY | 2 (0.3%) | | | | — | 4 (0.5%) | 3 (0.4%) | | 5 (0.7%) | | 2 (0.3%) |  | 15 (2.0%) | — | | — |
|  |  |  |  |  | 16 (2.1%) | | | | | | | | | | |  |  |  |  |  |
| 29 | M | CR | IV/  620* | Sanger/WAVE-based Surveyor | 1 (0.2%) | | | | — | 5 (0.8%) | 2 (0.3%) | | 17 (2.7%) | | |  | 20 (3.2%) | 2 (0.3%) | | 6 (1.0%) |
|  |  |  |  |  | [25 (4.0%)] | | | | | | | | | | |  | [28 (4.5%)] | | | |
| 33 | M | CR | I-IV/  513* | Pyro-  sequencing | 3 (0.6%) | — | | | — | 4 (0.8%) | 3 (0.6%) | | 7 (1.4%) | | 2 (0.4%) |  | 11 (2.1%) | 1 (0.2%) | | 5 (1.0%) |
|  |  |  |  |  | [19 (3.7%)] | | | | | | | | | | |  | [17 (3.3%)] | | | |
| 28 | NA | CR | I-IV/  376 | MALDI-TOF /Sanger | — | — | | | 1 (0.3%) | 1 (0.3%) | 1 (0.3%) | | 4 (1.1%) | | — |  | 16 (4.3%) | — | | 1 (0.3%) |
|  |  |  |  |  | [7 (1.9%)] | | | | | | | | | | |  | [17 (4.5%)] | | | |
| 34 | M | CR | I-IV/  224 | SOLiD/  Illumina HiSeq | 2 (0.9%) | — | | | — | — | 2 (0.9%) | | — | | — |  | 8 (3.6%) | — | | 1 (0.4%) |
|  |  |  |  |  | [4 (1.8%)] | | | | | | | | | | |  | [9 (4.0%)] | | | |
| 35 | NA | R | II-III/  94 | Sanger | — | — | | | — | 1 (1.1%) | 2 (2.1%) | | — | | — |  | 2 (2.1%) | 1 (1.1%) | | — |
|  |  |  |  |  | [3 (3.2%)] | | | | | | | | | | |  | [3 (3.2%)] | | | |
| 36 | 1 | CR | I-IV/  228 | Sanger | — | — | | | — | — | — | | — | | 1 (0.4%) |  | — | 3 (1.3%) | | — |
| 37 | NA | CR | IV/  84 | iPLEX/  Sanger | — | — | | | — | — | — | | 1 (1.2%) | | |  | 3 (3.6%) | — | | — |
| 38 | M | CR | IV/82 | Luminex | — | — | | | — | — | — | | 3 (3.7%) | | |  | 2 (2.4%) | — | | — |
| 39 | 1 | CR | I-IV/  220 | Sanger | 3 (1.4%) | | | | | | | | | | |  | 6 (2.7%) | 1 (0.5%) | | 2 (0.9%) |
|  |  |  |  |  |  |  |  |  |  |  |  |  |  |  |  |  | [9 (4.1%)] | | | |
| 40 | 1 | CR | I-III/  106 | PCR-RFLP | — | | | | | | | | | | |  | 7 (6.6%) | — | | — |
| 41 | NA | CR | I-IV/  205 | MALDI-TOF /MassARRAY | 4 (2.0%) | | | | | | | | | | |  | 7 (3.4%) | | | |
| 26 | 4 | CR | IV/ 76* | Pyro-  sequencing | 7 (9.2%) | | | | | | | | | | |  | 1 (1.3%) | | | |
| 42 | 1 | CR | NA/30 | HRM | 1 (3.3%) | | | | | | | | | | |  | 1 (3.3%) | | | |
| 43 | M | CR | IV/  400 | Pyro-  sequencing | — | | | | | | | | | | |  | 17 (4.3%) | | | |
| 44 | 1 | CR | NA/  30* | Pyro-  sequencing | — | | | | | | | | | | |  | — | | — | 1 (1.4%) |
| 45 | M | CR | IV/  711 | Pyro-  sequencing | 2 (0.3%) | 1 (0.1%) | | | — | 1 (0.1%) | 3 (0.4%) | | 9 (1.2%) | | 7 (1.0%) |  | Not examined | | | |
|  |  |  |  |  | [23 (3.2%)] | | | | | | | | | | |  |  |  |  |  |
| 46 | 1 | CR | I-IV/  672 | Sanger | — | — | | | — | 1 (0.2%) | 5 (0.7%) | | 8 (1.2%) | | |  | Not examined | | | |
|  |  |  |  |  | [14 (2.1%)] | | | | | | | | | | |  |  |  |  |  |
| 47 | 1 | CR | I-IV/  574 | Sanger | — | — | | | — | 1 (0.2%) | 4 (0.7%) | | 7 (1.2%) | | |  | Not examined | | | |
|  |  |  |  |  | [12 (2.1%)] | | | | | | | | | | |  |  |  |  |  |
| 48 | M | CR | IV/  284 | GS FLX sequencing | — | — | | | — | — | 1 (0.4%) | | 6 (2.1%) | | |  | Not examined | | | |
|  |  |  |  |  | [7 (2.5%)] | | | | | | | | | | |  |  |  |  |  |
| 49 | M | C | II-III/  239 | MALDI-TOF /OncoCarta | — | — | | | — | 1 (0.4%) | 1 (0.4%) | | — | | |  | Not examined | | | |
|  |  |  |  |  | [2 (0.8%)] | | | | | | | | | | |  |  |  |  |  |
| 50 | 1 | CR | I-IV/  133 | Sanger | — | — | | | — | — | — | | 1 (0.8%) | | 1 (0.8%) |  | Not examined | | | |
|  |  |  |  |  | [2 (1.5%)] | | | | | | | | | | |  |  |  |  |  |
| 51 | 1 | CR | NA/  304 | Pyro-  sequencing | — | 1 (0.3%) | | | 1 (0.3%) | 1 (0.3%) | 3 (1.0%) | | 4 (1.3%) | | |  | Not examined | | | |
|  |  |  |  |  | [10 (3.3%)] | | | | | | | | | | |  |  |  |  |  |
| 52 | 1 | CR | IV/  281 | Sanger | — | — | | | — | — | 2 (0.7%) | | 1 (0.4%) | | |  | Not examined | | | |
|  |  |  |  |  | [3 (1.1%)] | | | | | | | | | | |  |  |  |  |  |
| 53 | 1 | CR | IV/  143 | Sanger | — | — | | | — | 1 (0.7%) | 2 (1.4%) | | 1 (0.7%) | | |  | Not examined | | | |
|  |  |  |  |  | [4 (2.8%)] | | | | | | | | | | |  |  |  |  |  |
| 54 | NA | CR | NA/  130 | Sanger |  | 1 (0.8%) | | | — | 1 (0.8%) | 1 (0.8%) | | — | | — |  | Not examined | | | |
|  |  |  |  |  | [3 (2.3%)] | | | | | | | | | | |  |  |  |  |  |
| 55 | 4 | CR | IV/  111 | Sanger | — | — | | | — | — | 1 (0.9%) | | 2 (1.8%) | | |  | Not examined | | | |
|  |  |  |  |  | [3 (2.7%)] | | | | | | | | | | |  |  |  |  |  |
| 56 | M | R | LA/  82 | Sanger | — | — | | | — | — | 1 (1.2%) | | 1 (1.2%) | | |  | Not examined | | | |
|  |  |  |  |  | [2 (2.4%)] | | | | | | | | | | |  |  |  |  |  |
| 57 | M | CR | IV/  60 | Sanger | 1 (1.7%) | | | | — | — | — | | 2 (3.3%) | | |  | Not examined | | | |
|  |  |  |  |  | [3 (5.0%)] | | | | | | | | | | |  |  |  |  |  |
| 58 | 1 | CR | IV/  40 | Real time-PCR | — | — | | | — | — | 1 (2.5%) | | 1 (2.5%) | | |  | Not examined | | | |
|  |  |  |  |  | [2 (5.0%)] | | | | | | | | | | |  |  |  |  |  |
| 59 | 1 | CR | I-IV/  342 | Sanger | — | — | | | — | — | — | | 2 (0.6%) | | |  | Not examined | | | |
| 60 | 2 | CR | NA/68 | Sanger | — | — | | | — | — | — | | 1 (1.5%) | | |  | Not examined | | | |
| 61 | 1 | R | II-III/  37 | PCR-SSCP | — | — | | | — | — | — | | 1 (2.7%) | | |  | Not examined | | | |
| 62 | NA | R | NA/  118 | PCR-SSCP/  Sanger | — | — | | | — | — | — | | 2 (1.7%) | | — |  | Not examined | | | |
| 63 | 6 | CR | NA/  92 | Hybri-dization | — | — | | | — | — | — | | 1 (1.1%) | | — |  | Not examined | | | |
| 64 | 1 | CR | I-IV/ 65 | Pyro-  sequencing | — | — | | | — | — | — | | 1 (1.5%) | | — |  | Not examined | | | |
| 65 | NA | CR | I-III/  27 | Hybri-dization | — | — | | | — | — | — | | 1 (3.7%) | | — |  | Not examined | | | |
| 66 | 1 | CR | I-IV/  118 | Pyro-  sequencing | — | — | | | — | — | — | | — | | 1 (0.8%) |  | Not examined | | | |
| 67 | NA | CR | NA/43 | PCR-RFLP | — | — | | | — | — | — | | — | | 4 (9.3%) |  | Not examined | | | |
| 68 | M | CR | I-IV/ 101 | Sanger | — | 2 (2.0%) | | | — | — | — | | — | | — |  | Not examined | | | |
| 69 | 1 | CR | NA/53 | Infiniti assay | — | — | | | — | — | 2 (3.8%) | | — | | — |  | Not examined | | | |
| 70 | 5 | CR | IV/25 | Pyro-  sequencing | — | — | | | — | — | 1 (4.0%) | | | — | — |  | Not examined | | | |
| 71 | 4 | CR | I-IV/  381 | Sanger | 7 (1.8%) | | | | | | | | | | |  | Not examined | | | |
| 72 | 8 | CR | NA/  335 | PCR-SSCP/  DGGE | 8 (2.4%) | | | | | | | | | | |  | Not examined | | | |
| 73 | NA | CR | IV/150 | Cobas | 3 (2.0%) | | | | | | | | | | |  | Not examined | | | |
| 74 | M | CR | IV/68 | Pyro-  sequencing | 2 (2.9%) | | | | | | | | | | |  | Not examined | | | |

Cases missing *KRAS* status were excluded from a total number of cases of the study. (%) indicates the proportion of cases with a specific *KRAS* mutation among all cases analyzed successfully.

*, *KRAS* codon 61 and 146 mutations were analyzed only in cases that were *KRAS*-wild-type for both codons 12 and 13.

^†^, The raw data within this study were provided by personal communication (permission by Solit DB).

^§^, *KRAS* codon 146 mutations were analyzed only in cases that were *KRAS*-wild-type for codons 12, 13 and 61, and the details of *KRAS* codon 61 mutations were not provided.

When there were two or more specific types of mutation in one codon, the total number, and relative proportion of those mutations, are shown in square brackets.

ASP, allele specific primer; C, colon; CR, colon and rectum; DGGE, denaturing gradient gel electrophoresis; HRM, high-resolution melting analysis; LA, locally advanced; M, many; MALDI-TOF, matrix-assisted laser desorption ionization-time of flight mass spectrometry; NA, not available; PCR, polymerase chain reaction; R, rectum; RFLP, restriction fragment length polymorphism; SSCP, single strand conformation polymorphism
