# Supplementary material for: Analyses of clinicopathological, molecular, and prognostic associations of KRAS codon 61 and codon 146 mutations in colorectal cancer: cohort study and literature review
Source: Mol Cancer. 2014 May 31;13:135. doi: 10.1186/1476-4598-13-135 (PMC4051153; doi:10.1186/1476-4598-13-135)
Supplement: Additional file 7: Figure S1 — Pyrosequencing assay design and pyrograms for KRAS codons 61 and 146. [file 1476-4598-13-135-S7.pdf]

Figure S1

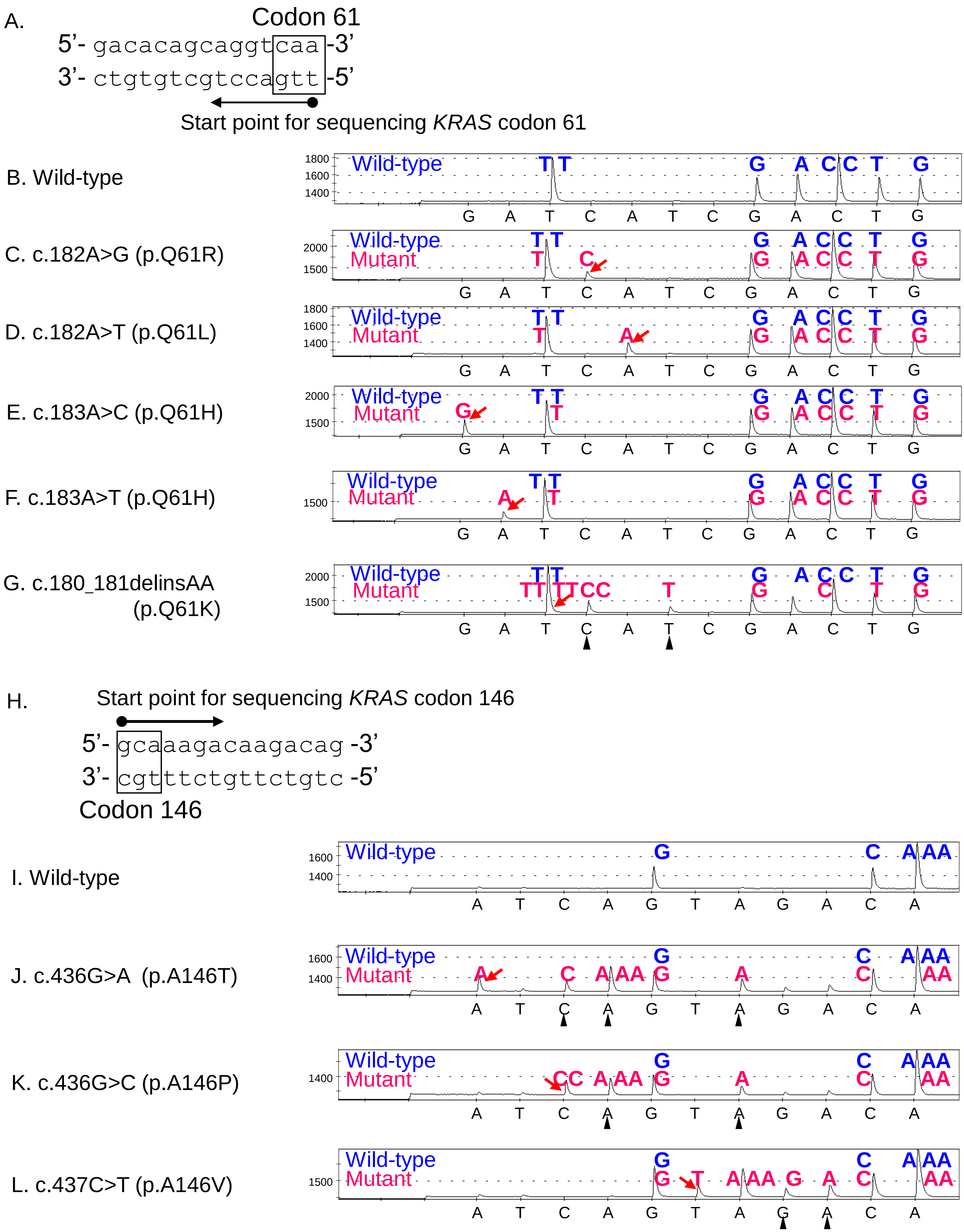

A.
Codon 61
5’- gacacagcaggtcaa -3’
3’- ctgtgtcgtccagtt -5’
Start point for sequencing *KRAS* codon 61
B. Wild-type
C. c.182A>G (p.Q61R)
D. c.182A>T (p.Q61L)
E. c.183A>C (p.Q61H)
F. c.183A>T (p.Q61H)
G. c.180_181delinsAA (p.Q61K)
Wild-type
Mutant
G A T C A T C G A C T G
H.
Start point for sequencing *KRAS* codon 146
5’- gcaaagacaagacag -3’
3’- cgtttctgttctgtc -5’
Codon 146
I. Wild-type
J. c.436G>A (p.A146T)
K. c.436G>C (p.A146P)
L. c.437C>T (p.A146V)
A T C A G T A G A C A
